# Self-Healing Concrete as a Prospective Construction Material: A Review

**DOI:** 10.3390/ma15093214

**Published:** 2022-04-29

**Authors:** Mugahed Amran, Ali M. Onaizi, Roman Fediuk, Nikolai Ivanovicn Vatin, Raizal Saifulnaz Muhammad Rashid, Hakim Abdelgader, Togay Ozbakkaloglu

**Affiliations:** 1 Department of Civil Engineering, College of Engineering, Prince Sattam Bin Abdulaziz University, Alkharj 16273, Saudi Arabia; 2 Department of Civil Engineering, Faculty of Engineering and IT, Amran University, Amran 9677, Yemen; 3 School of Civil Engineering, Faculty of Engineering, Universiti Teknologi Malaysia, Skudai 81310, Johor, Malaysia; mohammed.ahmed.ali@graduate.utm.my; 4 Polytechnic Institute, Far Eastern Federal University, 690922 Vladivostok, Russia; fedyuk.rs@dvfu.ru; 5 Peter the Great St. Petersburg Polytechnic University, 195251 St. Petersburg, Russia; vatin@mail.ru; 6 Department of Civil Engineering, Faculty of Engineering, Universiti Putra Malaysia, Serdang 43400, Selangor, Malaysia; raizal@upm.edu.my; 7 Department of Civil Engineering, Faculty of Engineering, University of Tripoli, Tripoli 13275, Libya; h.abdelgader@uot.edu.ly; 8 Ingram School of Engineering, Texas State University, San Marcos, TX 78666, USA; togay.oz@txstate.edu

**Keywords:** applications, self-healing, self-healing concrete, efficiency of self-healing, fiber, strategies, healing agent, bacteria, mechanism of self-healing

## Abstract

Concrete is a material that is widely used in the construction market due to its availability and cost, although it is prone to fracture formation. Therefore, there has been a surge in interest in self-healing materials, particularly self-healing capabilities in green and sustainable concrete materials, with a focus on different techniques offered by dozens of researchers worldwide in the last two decades. However, it is difficult to choose the most effective approach because each research institute employs its own test techniques to assess healing efficiency. Self-healing concrete (SHC) has the capacity to heal and lowers the requirement to locate and repair internal damage (e.g., cracks) without the need for external intervention. This limits reinforcement corrosion and concrete deterioration, as well as lowering costs and increasing durability. Given the merits of SHCs, this article presents a thorough review on the subject, considering the strategies, influential factors, mechanisms, and efficiency of self-healing. This literature review also provides critical synopses on the properties, performance, and evaluation of the self-healing efficiency of SHC composites. In addition, we review trends of development in research toward a broad understanding of the potential application of SHC as a superior concrete candidate and a turning point for developing sustainable and durable concrete composites for modern construction today. Further, it can be imagined that SHC will enable builders to construct buildings without fear of damage or extensive maintenance. Based on this comprehensive review, it is evident that SHC is a truly interdisciplinary hotspot research topic integrating chemistry, microbiology, civil engineering, material science, etc. Furthermore, limitations and future prospects of SHC, as well as the hotspot research topics for future investigations, are also successfully highlighted.

## 1. Introduction

Self-healing is a well-established and well-known property of concrete due to its innate autogenous healing properties [1]. After a period of time, fissures may mend due to the ongoing hydration of clinker minerals or carbonation of calcium hydroxide (Ca(OH)_2_). Autogenous healing, on the other hand, is restricted to minor cracks and is effective only when water is accessible, which makes it difficult to fully control or predict its accuracy. However, concrete can be adapted to include a bacterial stimulant system for sealing cracks [1]. Since the nineties of the last century, attempts began in the development of autonomous SHC [2]. In 2006, self-healing concrete (SHC) was invented as a new type of concrete by the microbiologist professor Henk Jonkers, at Delft University of Technology, Netherlands [3]. After 36 months of testing, he discovered the perfect healing agent, so-called bacillus.

**Figure 1:**
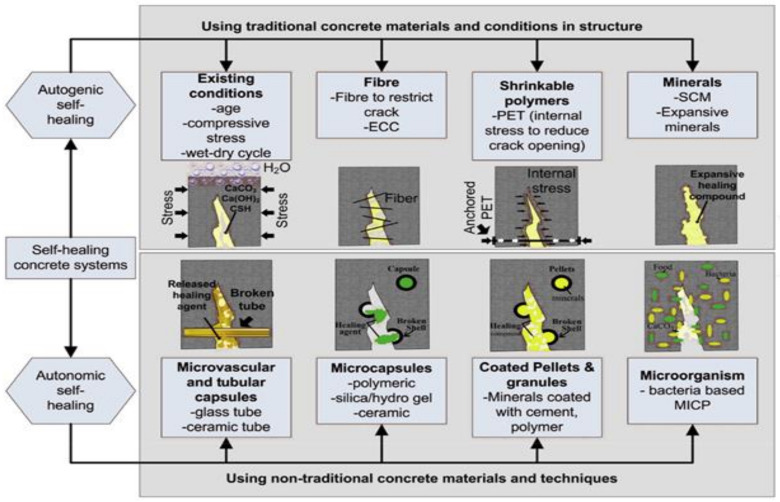
SHC systems (Adapted with permission from MDPI [4]).

SHC has a distinct system (Figure 1 [4]) and is commonly defined as concrete’s ability to heal cracks autogenously or autonomously [5]. It is also known as self-repairing concrete [6]. SHC imitates the automatic healing of body wounds by the secretion of some kind of material [7,8]. SHC is created by dispersing specific materials (e.g., capsules or fibers) containing repairing solutions into the concrete mix [9], where, when cracks appear, the fibers or capsules shatter, and the liquid contained within them spreads immediately to cure the crack. Concrete cracks are a common occurrence due to the low tensile strength of the concrete systems [10,11]. These developed cracks reduce the concrete’s long-term durability because they allow dangerous liquids and gases to leak through [1]. While concrete may be eroded by micro-cracks, steel reinforcement bars may also be affected by attacks resulting from the infiltration of harmful gases and liquids into the concrete system [2]. Therefore, in order to keep the cracks from widening, it is crucial that they are treated quickly. Self-healing of concrete cracks could extend the lifespan of concrete structures and make the structure more environmentally friendly while simultaneously increasing its sustainability [7].

Several self-healing strategies have recently been presented [1,2,7,9,10]. They primarily consist of self-healing methods such as capsule-based self-healing, vascular self-healing, electrodeposition self-healing [12], microbiological self-healing, and self-healing via shape memory alloy (SMA) integration [1,2,7]. It is worth mentioning that it has been claimed that early concrete exhibits the best capacity for self-healing [10]. To produce self-healing of concrete under pressure and cleavage, urea–formaldehyde microcapsules (20–70 μm in diameter) filled with epoxy resin and gelatin microcapsules (125–297 μm in diameter) filled with acrylic resin are utilized [13]. This is constructed of an air-curing agent that is delivered through glass tubes. The tubes are exposed to the atmosphere at one end and bent at the other to deliver the healing factor [14]. When the tubes’ content is exhausted as a result of concrete cracking, an extra chemical might be introduced through the open end to allow for the healing of larger cracks [15]. The electrode position approach is offered as a way for repairing fractured concrete structures, and the influences of this method on various concrete properties have been investigated [12]. Additionally, the ability of bacteria to act as a self-healing agent in concrete is explored, i.e., their ability to mend existing cracks. Authors demonstrated that using bacterial spores as a self-healing agent appears to be a potential application [16]. It is revealed that using SMA wire as a reinforcing bar can help to close cracks and repair emergency damage in concrete structures. Due to the highly elastic properties of inserted SMAs, the fissures are sealed [17].

One of the primary mechanisms underlying autogenic self-healing is the hydration of residual unhydrated cement in the matrix [4]. On the other side, this procedure produces a finite amount of healing products. As a result, autogenic self-healing is effective for cracks with a width of 50–150 μm [18]. In this situation, autogenic self-healing is highly enhanced in the early stages due to the presence of unhydrated cement, and characteristics such as compressive stress [19] to limit fracture propagation and wet–dry cycles can enhance the healing performance [20]. Autogenous healing performance can also be improved by utilizing fibers to limit crack progress, and using a superplasticizer in engineered cementitious composites (ECC) to minimize the w/c ratio, leading to the minimization of the probability of micro-crack generation [20,21]. A study team at Cardiff University used shrinkable polyethylene terephthalate (PET) tendons [22], which were activated using a heating system inside the concrete structural element to compress and fix the crack, accelerating the autogenous healing process. It is also feasible to significantly improve self-healing performance by utilizing optimal supplemental cementitious materials (SCMs) and smart expanding minerals [23,24,25,26,27,28,29,30]. In contrast to autogenous healing, autonomic self-healing in concrete necessitates the release of the healing agent from restricted encapsulation or a continuous vascular network. Some of the most common materials for encapsulation include glass [31,32] and polymers [33,34,35]. In addition, autonomic self-healing agents include bacteria-based microorganisms [16,36], alkali–silica solutions [13,31,32,37], methyl methacrylate [13,32], expansive minerals [38,39], and hydrogel [40].

There are several approaches used to evaluate SHC’s performance, including visual inspection and microstructural analysis, strength recovery, and enhanced durability [4]. In other words, self-healing can be evaluated in three ways: visual crack sealing monitoring and the identification of the healing compounds that cause it, improvement in durability performance, and recovery of strength properties [23,29,38]. However, strength recovery of concrete within the self-healing process is generally limited [41,42]. Therefore, self-healing behavior is most reliable when physical crack closure is observed, and durability improvement, i.e., permeability reduction parameters, and microstructural evaluations are conducted [30]. Therefore, there has been a surge in interest in self-healing materials, particularly self-healing capabilities in green and sustainable concrete materials, with a focus on different techniques offered by dozens of researchers worldwide in the last two decades. However, it is difficult to choose the most effective approach because each research institute employs its own test techniques to assess the healing efficiency. SHC has the capacity to heal and lowers the requirement to locate and repair internal damage (e.g., cracks) without the need for external intervention. This limits reinforcement corrosion and concrete deterioration, as well as lowering costs and increasing durability. However, this study reviews the strategies, influential factors, mechanisms, and efficiency of self-healing. This literature review also provides critical synopses on the properties, performance, and evaluation of the self-healing efficiency of SHC composites. In addition, we review trends of development in research toward a broad understanding of the potential application of SHC as a superior concrete candidate and a turning point for developing sustainable and durable concrete composites for modern construction today. Further, it can be imagined that SHC will enable builders to construct buildings without fear of damage or extensive maintenance. Based on this comprehensive review, it is evident that SHC is a truly interdisciplinary hotspot research topic integrating chemistry, microbiology, civil engineering, material science, etc. Moreover, this study highlights the limitations and research topics for further research as investment, as well as the future prospects in respect to SHC technology.

The article has the following logical sequence. After the Introduction, the Section 2 is devoted to SHC strategies. The Section 3 characterizes the factors influencing self-healing (moisture, crack width, hydration time, crack pressure, and water/cement ratio). The Section 4 describes the effectiveness of self-healing. The Section 5 presents the detailed mechanism of self-healing (autogenic self-healing and offline bacteria-based self-healing). The Section 6 is dedicated to SHC performance. In the Section 7, the effectiveness of self-healing is assessed (behavior at break, strength and survivability of the microcapsule shell, strength recovery). The Section 8 details the scope of the SHC. The Section 9 presents limitations and hot topics for future research. The Section 10 presents future prospects in more detail. Finally, the Section 11 is the final and generalizing one.

## 2. Strategies of SHC

**Figure 2:**
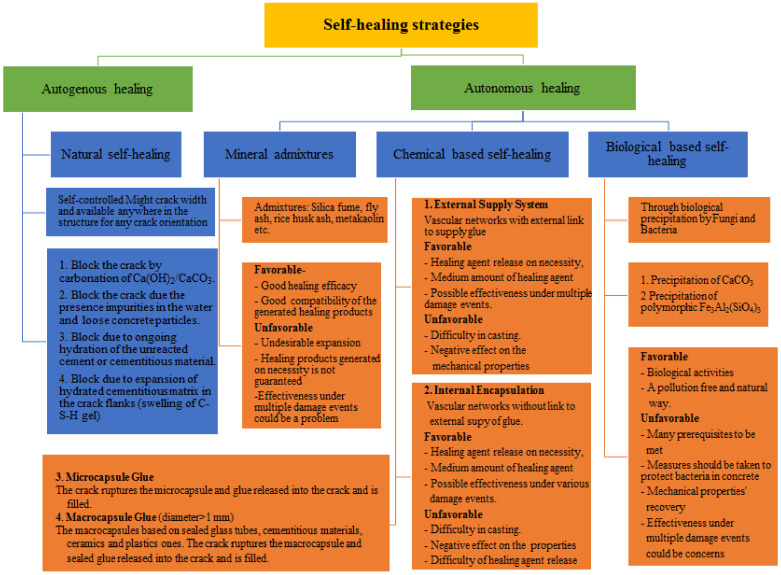
Strategies of self-healing phenomenon for concrete (Adapted with permission from IOP [43]).

SHC is developed by incorporating specific elements (such as fibers or capsules) into a concrete mix that contains repairing solutions. When a crack appears, the fibers or capsules break, and the liquid contained within them spreads and heals the crack at the same moment. Figure 2 illustrates concrete self-healing mechanisms [43].

Even though too many hollow fibers or capsules have a negative influence on the strength qualities of the cement matrix, several studies clearly reveal the possibility of self-healing under numerous damage events via encapsulation techniques. In this review study, an overview of self-healing techniques based on microbiological calcium carbonate production is presented. Furthermore, numerous obstacles associated with crack treatment by microbiological factors are explored, as well as providing recommendations for future study areas. Aside from bio-based healing and concrete durability, the cost of producing bio-concrete is also an issue. A more detailed examination of ways to lower costs related to bio self-healing, such as bacteria and nutrient costs is required [44]. Strategies to improve bio self-healing efficiency and lower prices will undoubtedly persuade builders to embrace bio-concrete as the material of choice in the near future. This material is quite new and little studied, so it is not yet regulated in most countries in the world. It is necessary to develop a special policy and create an extensive range of international regulations on self-healing concrete.

## 3. Influential Factors of Self-Healing

**Figure 3:**
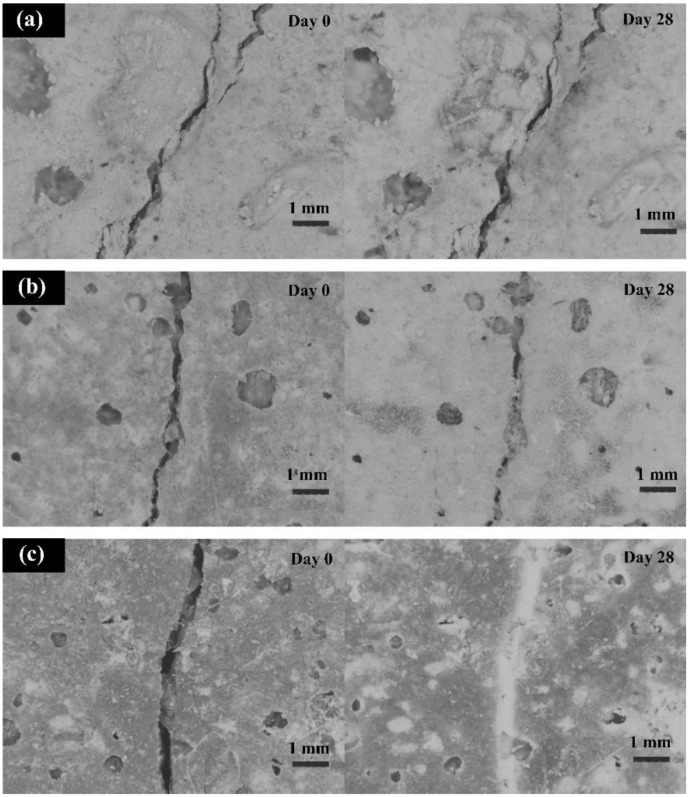
Evolution of crack healing via microbial process: (**a**) reference; (**b**) abiotic control; (**c**) microbial (Adapted with permission from Elsevier [45]).

The self-healing process is affected by a series of variables, including the healing agent used, the protective casing material, the dosage of capsules, the capsule diameter, the crack width and depth, the causes of cracks, the temperature and humidity, the concrete or mortar mix design, and finally the healing time. Figure 3 shows direct stereomicroscopic observation of cracks of bacteria-based specimens before and after complete healing [45]. The period that had been taken to heal cracks of 1 mm in width was 28 days of curing in tap water (Figure 3a). Small amounts of precipitates were found in the cracks of specimens with the addition of nutrients after incubation (Figure 3b). 

The appearance of the specimens with the addition of both spores and nutrients differed greatly. Not only were the cracks completely filled, but the surface of the specimens was partly covered by white precipitates after 28 days of healing. Some pores were also filled by the deposits (Figure 3c). The mechanism of crack healing is to produce calcium carbonate, which may subsequently be used to fill cracks [46]. During the self-healing process, calcium carbonate can be produced in two ways.

The first is that unreacted cement particles are employed to initiate hydration and form CaCO_3_. The second is that CaCO_3_ is generated during Ca(OH)_2_ dissolution [47]. There are several stages for generating calcium carbonate in different pH levels of water, as shown in chemical Equations (1)–(3) [47]. Previous studies have identified several factors that may impact a person’s ability to self-heal. The most important factors are listed below.

H_2_O + CO_2_ ↔ H_2_CO_3_ ↔ H^+^ + HCO_3_^−^ ↔ 2H^+^ +CO_3_^2−^ (1)

Ca^2+^ + CO_3_^2−^ ↔ CaCO_3_ (pH _water_ ˃ 8) (2)

Ca^2+^ + HCO_3_^2+^ ↔ CaCO_3_ + H^+^ (7.5 < pH _water_ < 8) (3)

### 3.1. Moisture Content

The availability of free water is critical to the healing of a cracked surface. Several previous studies have indicated that the presence of water plays an important role in facilitating crack healing. However, when the healing process is only efficient in the presence of water, the self-healing process has more difficulty to control its occurrence and it is also difficult to predict its efficacy [48,49]. It is reported that water accelerates the hydration of unhydrated cement particles and increases the dissolution of calcium hydroxide from the concrete matrix near the crack surface, resulting in the creation of calcium carbonate healing products. Another study indicated that the amount of available water in cracks and the time required for its diffusion are critical for efficient healing [35]. In general, it was found that cracks mend themselves with the assistance of continuing calcium-silicate-hydrate gel formation and calcium carbonate precipitation processes and the existence of air and water as well [50]. Water is described as the most important environmental component in engaging ECC healing in terms of the cementitious matrix as a means of reducing crack growth [51]. Water curing was also reported to be the optimal environment for bacteria-based SHC [52].

### 3.2. Crack Width

In general, spontaneous or autogenous healing is most efficient for extremely small cracks of less than 0.3 mm in width [53,54] (see Table 1). The potential of self-healing strategies to properly and efficiently close crack widths is still a major concern. To date, *Bacillus sphaericus* can completely heal a maximum crack width of 0.97 mm, which is almost four times that observed in a non-bacteria sample [35]. For the control group, the average width and maximum width of cracks that could be repaired were around 36 µm and 56 µm, respectively [55]. However, cracks wider than 0.3 mm in width may not repair. However, cracks with a width of 0.1 mm are entirely healed within 200 h. In addition, cracks between 0.2 and 0.3 mm in width often heal within 30 days [56]. Furthermore, cracks between 0.15 and 0.3 mm in width dramatically reduce in 7 days and completely heal within 33 days [24]. According to several reports, the greater the width of the crack and the greater the number of cracks, the greater the volume of the self-healing product produced; however, if the width of the crack is relatively large, the self-healing products become insufficient to fill the large volume due to the effect of the width of the crack itself [57]. From a microscopical perspective, the self-healing products appear to be crisscrossed and spread in a spatial network, effectively plugging fractures and pores. Furthermore, it seems that the self-healing products not only aid in mending, but they also serve as an efficient waterproofing agent. On the other hand, it was reported that the repair impact decreased as the cracking age increased and that the age of the crack as well crack width had a significant effect on self-healing performance [58]. In general, the process of self-healing of cracks in hardened concrete is dependent on the inclusion of agents such as minerals, bacteria, and microcapsules containing adhesive elements in the cementitious compositions [59]. The challenge is when it comes to concrete building in marine settings such as seashores; such aggressive environments raise a number of issues owing to the biological and chemical behavior of the surroundings. These data might be utilized to demonstrate that self-healing cementitious materials can significantly extend the service life of buildings in chloride-containing environments [60]. Therefore, the healing capacity (H%) can be calculated based on the relevant difference between the strength reduction (h^0^) of the reference specimens after damage and the strength recovery (h^1^) of the damaged specimens after healing (Equation (4)) [61].

H = (h^0^ − h^1^)/h^0^ (4)

The durability of SHC can be computed through the measurement of the chloride migration penetration depth, measured from the visible white silver chloride precipitation, expressed (Equation (5)) as the non-steady-state migration coefficient [62].

D_nssm_ = (0.0239 × (237 + T) × L)/(U − 2) × t) + [x_d_ − 0.0238 ((237 + T) × L × x_d_)/(U − 2))^0.5^ (5)

where

- D_nssm_ is the non-steady-state migration coefficient [m^2^/s];
- U is the absolute value of applied voltage [V];
- T is the average value of the initial and final temperatures in the analyzed solution [K];
- L is the thickness of the specimen [m], and
- x_d_ is the average value of the penetration depth [m]. Further, the following Table 1 summarizes the role of bacteria in crack healing.

**Table 1:** The performance of bacteria species in healing cracks.

Type of Bacteria	Type of Healing Agent	Embedded	Strength Recovery	Width of Crack, mm	Durability Effect	Refs.
*Subtilis*	Urea, CaCl_2_ H_2_O	Diatomite lam dong	x	1–1.8	√	[63]
*B. Pseudomycoides*	Ureolytic activity	Directly with 100 mL cell	√	0.15–0.3	√	[64]
*Subtilis*	Urea—2CaCl_2_ curing	Directly with 2.2 × 10^6^ cells/mL	x	0.2	√	[65]
*Sporosarcina pasteurii*	Urea—CaCl_2_ curing	Directly with 10^7^ cells/cm^3^	√	0.28–0.34	√	[66]
*Sphaericus*	Urea, yeast extract, Ca(NO_3_)_2,_ 4H_2_O	Diatomaceous earth with 10^9^ cell/mL	x	0.15–0.17	√	[67]
*B*. *Megaterium*	Urea yeast extract, beef extract	Directly with 2.2 × 10^6^ cells/mL	√	0.3	√	[68]
*Sphaericus*	Urea, yeast extract, Ca(NO_3_)_2,_ 4H_2_O	Hydrogelencapsulated spore	x	0.5	√	[69]
*B*. *Subtilis*	Urea CaCO_3_ crystals, yeast extracts, NaCl	Steel bar, Hach dr 2400 portable	√	1.-	√	[70]
*Sphaericus*	Urea, Ca(NO_3_)_2_, 4H_2_O	Silica gel, polyurethane	√	0.35, 0.25	√	[71]
*Megaterium, licheniformi*	Urea-broth culture	Direct with 10^5^ cell/mL of mixing water	x	0.3	√	[72]
*Sphaericus*	Urea, calciumnitrate, yeast extract	Microcapsule	x	0.97	√	[35]
*B*. *Sphaericus*	Urea Ca^2+^ ion, CaCl_2_ usage	Trinocular stereomicroscope	√	0.4	√	[73]
*Pasteurii*	Mixing water was replaced by urea–yeast extract medium	Direct with 2–6 × 10^7^ cfu/mL	√	-	x	[74]
*Sphaericus*	Urea, Ca(NO_3_)_2_	Glass tubes with PU foam	√	0.3	-	[75]

Annotation: (√), negative effect, (x), positive effect.

### 3.3. Time for Hydration

**Figure 4:**
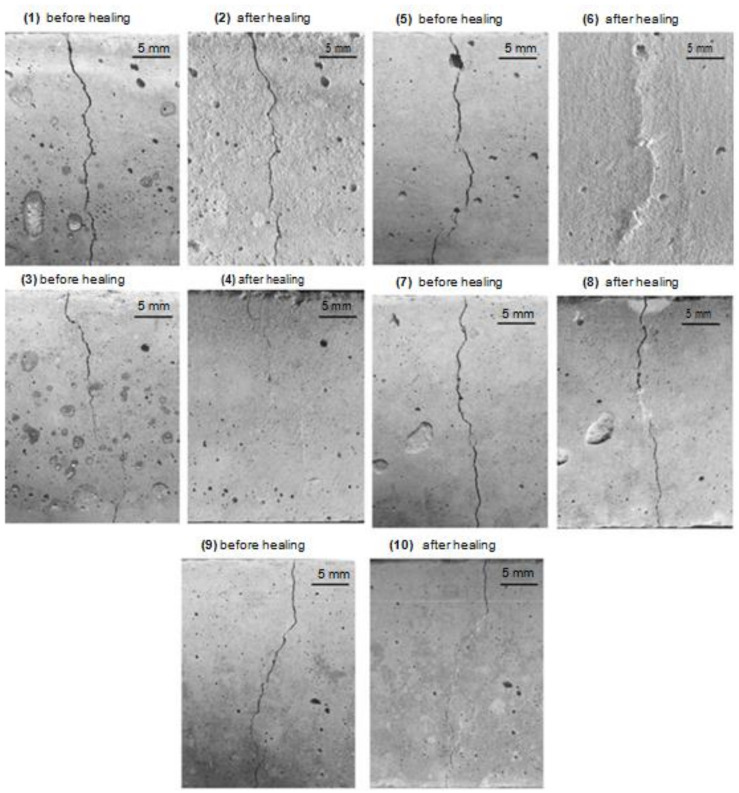
Images of crack healing process (Adapted with permission from Elsevier [76]).

It is widely established that prolonged hydration results in improved self-healing capacity. Self-healing is a well-established and well-known property of concrete due to its innate autogenous healing characteristics [10]. Figure 4 illustrates the phenomena of crack healing of the specimens before and after the healing of five different concrete mixes [76,77]. After a period of time, cracks could heal due to the ongoing hydration of clinker minerals. Autogenous healing, on the other hand, is restricted to minor cracks and is effective only when water is accessible, making it difficult to regulate. By controlling the w/c ratio and inclusion of several pozzolanic materials, concrete may be adapted to self-heal cracks [78]. It is reported that lowering the w/c ratio in cement-based concrete systems leaves an amount of unhydrated cement within the cement-based concrete matrix [79]. This unhydrated cement amount could contribute to autogenous healing once the crack develops in the concrete matrix, allowing water to penetrate and hydrate the remaining amount of unhydrated cement.

It seems that the hydration rate is significantly greater at low w/c ratios than it is at high w/c ratios. This is due to the fact that the hydration kinetics of cementitious composites containing mineral additives can be slow in the first weeks, so, in recent years, several methods have been proposed to stimulate and accelerate the self-healing process, including using alkaline solutions [80], and the mixing of various mineral additives [81], including calcareous fly ash [81], lime powder [82], or hydrated lime [82], to increase the calcium content. The age curing time of the specimens also is considered a factor that affects the degree of hydration of cement particles and other SCM. According to previous research on self-healing, this mechanism is particularly successful in fresh concrete. Although Zamorowski [83] demonstrated that the complete filling of cracks is only possible if they form during the initial stage of concrete hardening (during the first 90 h), composites have been developed over several decades that allow for the restoration of the structure’s original integrity many months after the start of hydration [84,85], and the effects of autogenous self-healing can be observed even in long-term structures [86]. In summary, the lower the degree of hydration of the binders used, the more substrates for the chemical reactions that accompany the self-sealing process remain in the composite structure; thus, composites with a lower degree of binder hydration and a lower w/b ratio should have higher self-healing potential.

### 3.4. Pressure Loaded on Cracks

SHC, also known as bio-concrete, can be produced by adding bacteria in concrete along with its nutrients to keep them alive for the production of calcite to fill cracks after precipitation. Bacteria are to be added in concrete along with calcium lactate to repair cracks. It was noted that the crack’s capillary attractive force and the gravitational force on the fluid mass were inadequate to overcome the cylindrical capsules’ capillary resistive force and the negative pressure forces created by the sealed ends [15]. However, the majority of previous studies have focused on the self-healing of concrete specimens subjected to typical water pressure when partially or completely immersed in water. A water permeability test was used in a limited number of studies as a means to emphasize the self-healing characteristic of normal concrete [87,88]. Likewise, it has been utilized on small concrete specimens that were subjected to single tension crack. After seven weeks of water exposure on unloaded specimens, the finding revealed that cracks with an effective width of 0.20 mm were entirely sealed [85]. Another study looked at water leakage through a pre-cracked RC element under direct tensile load, where the load was released immediately after cracking [89]. It was found that after unloading, the outer cracks shrank to 0.15 mm, culminating in entire healing after two days of exposure to water. In addition to the fact that ECC was not tested under high water pressure, previous studies on pre-cracked normal concrete under water pressure mainly investigated the self-healing on unloaded specimens [90]. However, as compared to the loaded condition of specimens, crack widths were demonstrated to fall by more than half upon unloading [91], which may have contributed to an overestimation of the self-healing capacity of standard concrete samples [92]. Furthermore, previous studies conducted to evaluate the influence of constant and increasing sustained loading revealed that continuous loading significantly affected the rate of recovery of the mechanical properties of ECC [93]. Hence, studying the effect of sustained loading while assessing the self-healing of concretes is particularly significant, particularly given that most cracked structural parts are subjected to creep processes in realistic field circumstances [94]. Generally, it is discovered that applying correct pressure on cracks boosts their potential to self-heal.

### 3.5. Water–Cement Ratio

**Figure 5:**
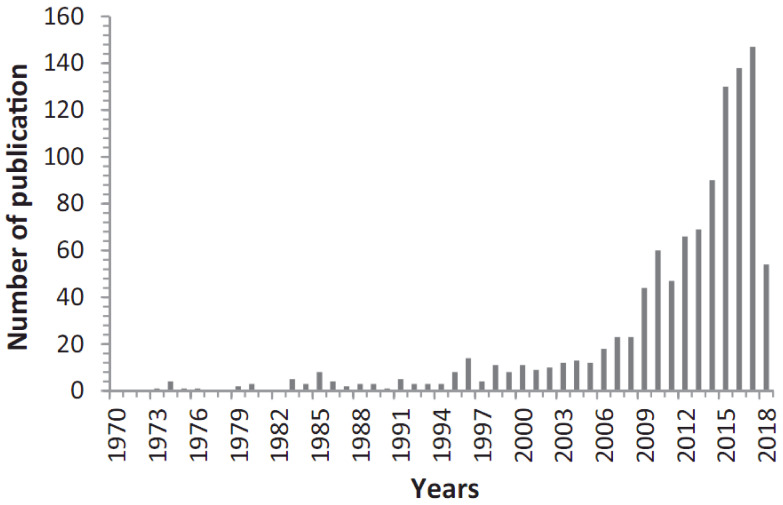
Previous studies concerning both concrete/mortar and cement pastes’ influence on self-healing, up to 2018 (Adapted with permission from Elsevier [96]).

It is found that the use of high cement content and a low water-to-cement ratio also increases the autogenous self-healing capacity of ECC [4]. According to previous reports, a lower water–cement ratio can result in more unreacted cement particles that can be employed for subsequent hydration to improve calcium carbonate production. Furthermore, the cracking time is significant. Because earlier-cracking concrete has more unreacted cement particles surrounding the developed crack, it provides higher self-healing potential as a result of boosting hydration continuation [46]. Due to the high percentage of unhydrated cement at early age, the autogenic self-healing capacity is greater, and other characteristics such as compressive pressure [95] to restrict cracks and wet–dry cycles [20] can boost the autogenous self-healing capacity. Moreover, the use of fibers to limit crack opening and the use of a superplasticizer in ECC to lower the w/c ratio can also improve autogenic healing efficacy [20]. It was reported that in the case of replacing cement with calcium sulphoaluminate pellets, up to 10 wt% of cement, and with a 1:3 cement-to-sand ratio and w/c = 0.5, the cracks between 0.1 and 0.2 mm were fully sealed in 14 days, but cracks larger than 0.2 mm took 16 days to fully seal. In summary, it has also been discovered that using high cement content and a low water-to-cement ratio improves ECC’s autogenous self-healing potential. However, fibrous concrete and ECC are considered relatively expensive, and maintaining fiber homogeneity in the matrix for constant self-healing still represents a challenge. Figure 5 depicts many self-healing groups, including autogenous and autonomous [96], as well as information on the material type, paste, mortar, or concrete [97,98]. This could enable us to determine whether researchers have considered the impact of mixture elements, such as aggregates. The percentages show the number of papers that cited the material type. The influence of coarse particles on the fracture pattern was overlooked, as demonstrated. This is largely because the aggregates will reduce the capsules’ survival throughout the mixing and transporting operations.

## 4. Efficiency of Self-Healing

Self-healing is believed to be promising in enhancing the durability and serviceability of cementitious structures, and it is widely understood that water is a crucial factor for the self-healing process to occur [99] (see Table 2). *Bacillus pasteurii* has also been shown to enable cracked concrete to regain 90% of its original strength. According to Victor Li of Michigan University [100], when the concrete is loaded again after it has healed, it acts almost exactly as new, with approximately the same strength and stiffness. Another study [101] reported that the natural self-healing in concrete can occur through four different processes: (1) precipitation of calcium carbonate or calcium hydroxide, (2) blocking of cracks by impurities in water, (3) continuation of hydration of unreacted cement or additive cementitious materials, and (4) expansion of hydrated products around the crack flanks (i.e., C–S–H swelling) [102]. It is also worth noting that calcium carbonate and calcium hydroxide precipitation represent the most efficient strategies for healing concrete cracks autogenously [101]. It was revealed that the healing effects during freeze–thaw cycles may have a major influence on durability [103,104]. Thus, several researchers [105,106,107] focused on self-healing action following freeze–thaw cycles [108]. Jacobsen et al. [109,110] reported self-healing of frost-damaged concrete beams and ascribed it to their test method: they performed the freeze–thaw cycle in water and found complete recovery of the resonant frequency in that situation. It is also emphasized that the self-healing process of concrete can be accelerated by allowing a 30-day rest period between freeze–thaw cycles, which facilitated self-healing, where the freeze–thaw tests were conducted in water, and the authors confirmed that the self-healing action increased the concrete’s frost resistance [106]. A slight improvement in performance was observed during a freeze–thaw cycle in a soil–cement specimen, which was ascribed to hydration and healing processes [107]. The self-healing behavior of pre-cracked designed cementitious composites that displayed strain-hardening behavior during freeze–thaw cycles using tensile, ultrasonic pulse, and sorptivity absorption tests was also evaluated [108]. The authors observed that the self-healing behavior was dependent largely on pre-loaded strain levels, where greater pre-loaded strain levels resulted in a decreased capacity for self-healing [108].

In recent years, researchers have reported the self-healing behavior of ultra-high-performance fiber-reinforced concrete (UHPFRC), which has potential for self-healing because the incorporated micro steel fibers restrict cracking, following cryogenic cooling [134,135]. A simple approach (i.e., applying cryogenic cooling) for self-healing pre-existing UHPFRC constructions is possible, where the UHPFRC beams are saturated due to water from melting frost that develops on the beam surface. However, very few researchers [132,136] have investigated the self-healing behavior of UHPFRC. Granger et al. [132] used flexural and acoustic emission experiments to investigate the self-healing capacity of pre-cracked ultra-high-performance concrete (UHPC) without steel fibers. They also looked at the influence of the re-curing regime on self-healing behavior. The researchers discovered that pre-cracked (damaged) beams retain their initial stiffness and freshly formed crystals develop only after re-curing in water. Furthermore, the researchers observed that re-curing in air results in no recovery or crystal growth [132]. The influence of self-healing on the micro-cracks and air permeability of pre-cracked UHPFRC was explored by Kwon et al. [136], who revealed the precipitation of calcium carbonate (CaCO_3_) inside micro-cracks and the recovery of air permeability during water re-curing. In comparison to other types of concrete and fiber-reinforced concrete, research into the self-healing behavior of UHPFRC is still in its early stages, with just one study [134] looking at the self-healing capabilities of UHPFRC following exposure to cryogenic temperatures in a liquefied natural gas tank application.

## 5. Mechanism of Self-Healing

### 5.1. Autogenic Self-Healing

**Figure 6:**
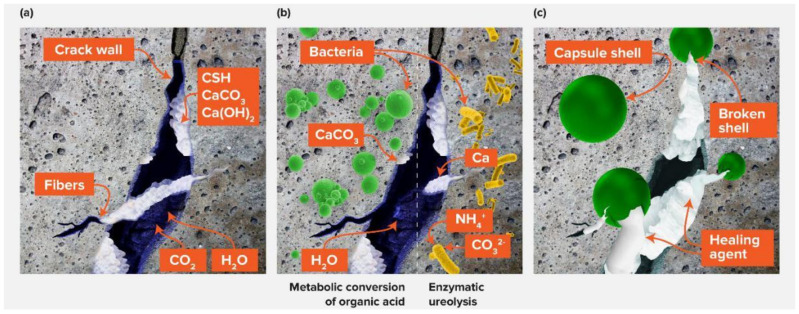
Self-healing mechanisms: (**a**) autogenous, (**b**) autonomous bacteria-based, and (**c**) autonomous capsule-based (Adapted with permission from IOP [139]).

Autogenic self-healing (ASH) is the ability of concrete to repair or heal cracks in the presence of moisture and the absence of tensile stress [137]. The utilization of concrete’s self-healing characteristic is critical, especially for water-retaining buildings, since it is a brittle material that changes dimensions based on the quantity of moisture present within [47]. In the concrete sector, the healing processes are typically categorized into two categories: autonomic and autogenous healing [33,138] (Figure 6) [139]. The main function of the self-healing process is sealing cracks in the concrete structure, thus extending the service life of concrete structures. This would also make the structures more durable, and also more sustainable [29]. The autogenic self-healing process uses only original material components that promote healing due to their distinct and active chemical nature under favorable environmental circumstances for this process to occur [33]. At the beginning of the nineteenth century, the phenomenon of autogenic self-healing began to attract the attention of researchers since the first water-retaining structures, culverts, and pipes were observed to heal themselves [53]. Later studies focused on the determination of the physicochemical background of this process [140,141,142,143]. Generally, the reasons for autogenous self-healing may be classified into three categories: physical, chemical, and mechanical [33]. Physical factors include expansion of the cement matrix around the crack opening as a result of water absorption by the hydrated cement matrix, leading to the narrowing of the crack. The chemical reasons are due to two primary processes: the continuing hydration of Portland cement and the precipitation of calcium carbonate around the cracks (see Figure 4). The mechanical effect of autogenic self-healing is the filling of opened cracks with small particles seeped from the damaged concrete surface or transferred to the crack by water [33].

**Figure 7:**
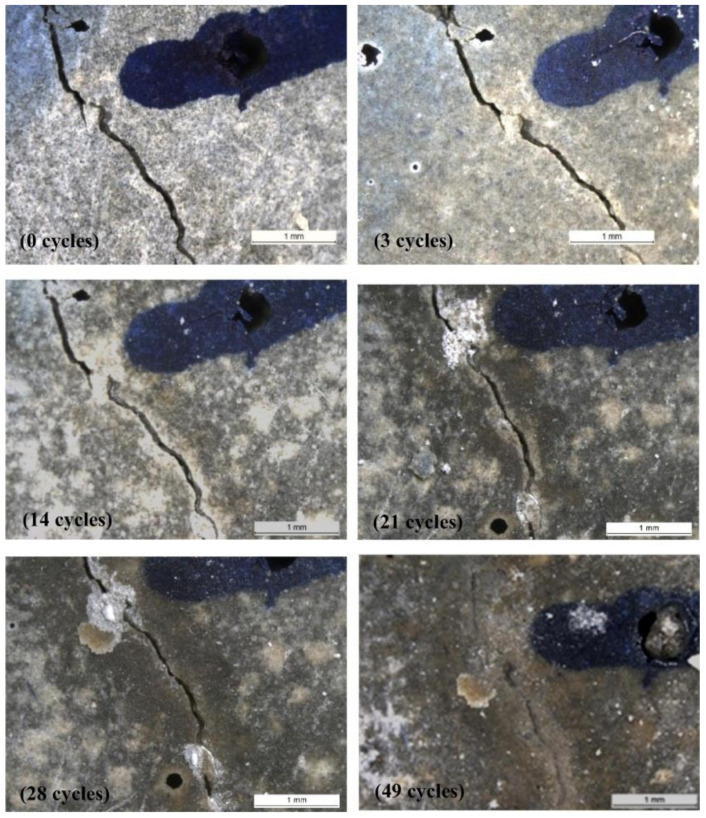
Closure of a 100 μm crack in an OPC M (0.45) sample cyclically exposed to 33 g/L NaCl (Adapted with permission from Elsevier [145]).

The marine environment has an impact on autogenous self-healing as well [144,145]. Ordinary Portland cement (OPC) mortar specimens outperformed blast-furnace slag (BFS) mortar specimens in a laboratory test simulation with saltwater immersion [144]. In freshwater, however, the situation was inverted. After 56 days, 100% OPC and a BFS mixed OPC sample in seawater sealed cracks up to 592 µm and 104 µm, respectively, but in freshwater, the situation changed to 168 µm and 408 µm. The precipitation of brucite, i.e., Mg(OH)_2_ and aragonite, was also observed to have a significant impact on the healing process. In a another study [145], and to generate more knowledge about the efficiency of autogenous self-healing in marine conditions, the autogenous healing effectiveness of OPC and BFS mortar samples in chloride and a mixture of chloride and sulfate solution to simulate a marine environment was studied (see Figure 7) [145]. The finding revealed that while a chloride solution had no effect on the healing process, solutions containing extra magnesium sulfate increase crack sealing by creating brucite layers at the crack planes. As a result, it can be concluded that the cement matrix compositions, such as certain supplementary cementitious materials and the healing environment, have a substantial impact on the autogenous healing process.

Reportedly, the use of pozzolanic minerals was proven to improve the autogenous self-healing capacity of OPC concrete when used as supplementary cementitious materials due to their pozzolanic activity [23,24]. The optimal pozzolanic reaction of supplementary materials promotes the formation of effective hydration products that are capable of not only sealing but also healing cracks [38,146,147]. Table 3 summarizes published research on the use of mineral additives to enhance autogenous self-healing. Various researchers have used calcium sulfoaluminate, lime, bentonite clay, fly ash (FA), silica fume (SF), and blast-furnace slag (BFS), and the table shows all minerals in this regard. Table 3 shows the development trends on autogenous self-healing concretes with the addition of mineral additives.

### 5.2. Autonomic Bacteria-Based Self-Healing

**Figure 8:**
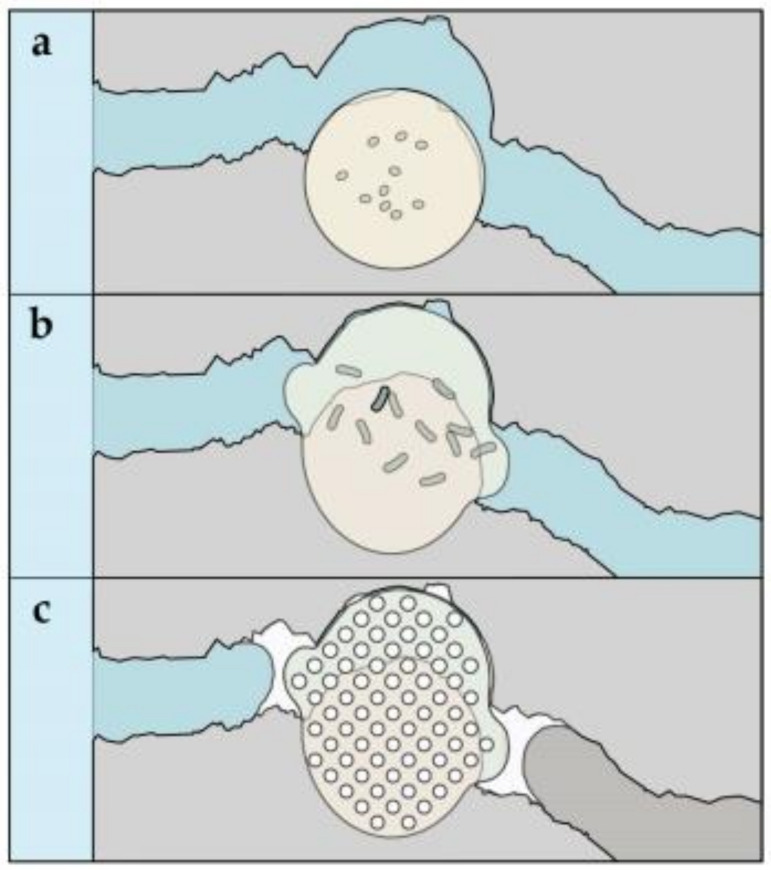
Schematic diagram illustrating the proposed healing mechanism: (**a**) in the event of cracking and water ingress; (**b**) the bacteria-based beads incorporated in the composite will swell, this swelling will clog the cracks, and concomitantly “free up” the bacteria, yeast extract and magnesium acetate contained in the beads; (**c**) the magnesium will precipitate as magnesium-based minerals, the spores will germinate as a result of being exposed to the solubilized yeast extract, and metabolize the acetate, inducing calcium-based mineral precipitation in and on the surface of the beads, healing the crack. (Adapted with permission from MDPI [161]).

The autonomic self-healing mechanism relies on additives such as microcapsules containing healing capsules filled with bacteria spores, which are considered responsible for depositing the healing materials [3,159,160]. Autonomic bacteria-based self-healing is an alternate approach that frequently employs bacteria in the form of spherical thick-walled cells, such as alkaliphilic endospore-forming bacteria [119]. Jonkers was the first to introduce a bacteria-based SHC agent composed of bacterial spores and an organic mineral precursor chemical [3]. In these systems, the self-healing mechanism is dependent on the synthesis of calcium carbonate by the bacterial metabolic conversion of organic acids or enzymatic ureolysis. However, in this mechanism, the self-healing process occurs only in the presence of an organic substrate that provides nutrients and water for bacteria to stimulate the activity of bacteria [161]. Crack-induced water ingress activates bacterial spores, which mature into active organic cells capable of converting mineral precursor chemicals to calcium carbonate. Calcium carbonate precipitated in steel-reinforced concrete cracks can prevent water infiltration through the cracks and minimize susceptibility to chloride intrusion and thus reduce the probability of steel reinforcement corrosion. Despite the fact that the majority of the world’s marine infrastructure is located in mild climate zones (annual average temperature of 10 °C, and average summer temperature of 20 °C) [162], bacteria-based SHC has been demonstrated to occur in better in freshwater and room-temperature conditions [3,16,35,56,69,119,163]. If bacteria-based SHC is to be realized in low-temperature marine environments, the bacteria-based agents employed to create SHC must also operate in such environments. Furthermore, it has been demonstrated that effective microorganisms injected directly into concrete after mixing have limited functioning over time due to a lack of nitrates [16]. As a result, and to protect bacteria-based agents in such conditions, bacterial spores were encapsulated in expanded capsules of clay [56,119], and bacteria in diatomaceous earth [67] and melamine-based microcapsules [35,164], before synthesizing them into the cementitious composites. Although these tactics extend the time period for microorganism activity to attain healing, they do not provide a remarkable increase in repair ability. Alginates have recently been proposed as a protective transporter of bacterial spores [165] and for the production of a bacteria-based bead [166]. Based on the latter study, the bacteria-based bead, which is composed of bacterial spores encapsulated in calcium alginate, which contain a bacterial nutrient source (yeast extract) and a mineral precursor compound (magnesium acetate), swelled when submerged in a low-temperature (8 °C) simulative marine concrete crack solution, forming a bacteria-activated calcite (CaCO_3_)–alginate composite material [161]. It is anticipated that incorporating this bacteria-based bead technology into a cementitious material would grant it superior crack-healing activity. In Figure 8, a schematic of the proposed healing mechanism for a cementitious composite incorporated with the bacteria-based bead technology is provided [161]. In the case of breaking and water ingress (Figure 8a) [161], beads along the crack would expand, blocking the crack (Figure 8b); this swelling of beads frees up the bacterial spores, yeast extract, and magnesium acetate. Releasing the beads’ content will result in the magnesium acetate precipitating as magnesium-based minerals, and the spore organisms will activate as a result of their exposure to the solubilized yeast extract. Their metabolization of the acetate, inducing calcium-based mineral precipitation in and on the surfaces of the bacteria-based beads, thus contributes to healing the crack (Figure 8c) [161]. In brief, the bacteria-based self-healing cementitious composite holds great promise as a cost-effective self-healing material in low-temperature marine environments. Furthermore, the organic–inorganic composite healing concrete technology represents an attractive direction for self-healing concrete research (see Table 4).

### 5.3. Autonomic Capsule-Based Self-Healing

The autonomic self-healing process is based on the action of additives such as healing-agent-filled microcapsules [159,160]. One of the most prominent strategies researched in recent years is the encapsulation of a healing substance. The sealing action takes place once a crack has begun inside the binder matrix and propagates across a capsule, breaking it, and releasing the healing agent. The healing substance that has been released then seals the fracture and prevents it from spreading further. The permeability of the concrete matrix usually is reduced as a result of reducing the crack width and crack ratio, and there is typically a partial recovery of mechanical qualities [167]. Typically, capsules comprise urea–formaldehyde, glass, and silica, and calcium nitrate, epoxy resin, polyurethane, and superabsorbent polymers (SAP) are considered the most frequent forms of healing agents [97,168]. In this regard, Nishiwaki et al. [115] reported that the encapsulation of healing agents is a very effective method for obtaining complete crack sealing with chemical agents, avoiding aggressive substance penetration, and, in some cases, obtaining partial mechanical property restoration, which is an important aspect of ensuring performance during service life (succession of crack formation).

**Figure 9:**
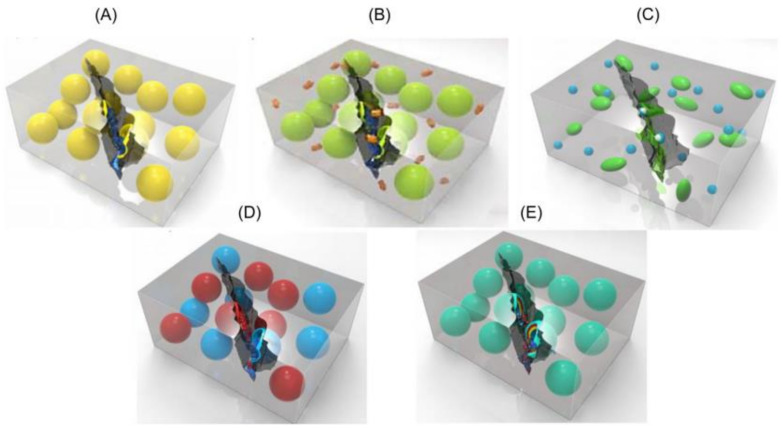
Capsule-based self-healing systems: (**A**) single capsules, (**B**) capsule (green)/dispersed catalyst (orange), (**C**) phase-separated droplet/capsules (green), (**D**) double-capsule (blue capsules with hardener, red capsules with healing agent) and (**E**) all-in-one microcapsules (multiple shell walls depicted with different colors) (Adapted with permission from Elsevier [170]).

The design cycle for capsule-based self-healing materials involves the following steps: (1) capsule encapsulation; (2) capsule integration into the matrix; (3) mechanical characterization; (4) healing agent triggering and release to the damaged region; and (5) healing agent assessment [169]. Five distinct types of encapsulated healing agent systems have been demonstrated to be effective and are addressed in further detail in the following sections (see Figure 9) [170]. At least one healing agent is encapsulated in the single-capsule system, which can be a reactive chemical, a solvent, or a low-melting-point metal.

The capsule/dispersed catalyst healing mechanism is based on encapsulating a self-healing agent within brittle capsules and dispersing the catalyst/hardener within the matrix. Damage propagating in the crack form results in the capsules breaking and releasing the monomer, which then comes into contact with the catalyst, resulting in polymerization [171]. The third strategy is a phase that separates droplet/capsule systems, involving the phase separation of at least one of the healing components and encapsulation of the remaining component. When these two substances are released, they react with one another. While the double-capsule system incorporates one or more reactive liquid healing agents or polymerizers, the all-in-one microcapsule system is completely self-contained [169,171]. However, glass capsules have usually been employed, primarily to keep the healing agent [31,37,122,172]. These capsules break upon crack appearance satisfactorily, but the challenge comes from the fact that they are unable to withstand the concrete mixing process without special protection [173]; thus, researchers have suggested bundling the capsules with a water-soluble solution, which could protect them during the truck mixing process [174]. Table 5 summarizes the encapsulation strategies employed in various studies.

This is explained by their brittleness and size: glass tubes with a wall thickness of 2 mm can rupture upon crack development, whereas tubes with a wall thickness of 3 mm cannot burst satisfactorily upon crack formation [175,176]. The continued development of mixing techniques that would maintain the survival of the healing agents during the mixing process would enhance the use of self-healing agents for concrete, reduce its cost, and enhance the confidence of the stakeholders in the construction sector with this technique. A shift toward polymeric encapsulating materials may provide a means of more readily adjusting the capsule characteristics and resisting the mixing process, as suggested by Dry [177]. Apart from glass, other materials such as natural fibers [178], gelatin capsules [159], paraffin to enclose water [179], and polyurethane [124] have been used. Ceramic, a highly fragile material, has also been employed, and a stronger connection than with glass was found [115]. While spherical capsules can be successfully combined with concrete [34,119,124,159,173], cylindrical capsules have a higher probability of breaking upon crack development [173] and release the healing agent more efficiently, even if their orientation is still a concern [180]. Polypropylene capsules covered with wax and heated to release the contents have been explored in order to develop capsules that can withstand the concrete mixing process [2].

**Table 5:** Details of encapsulation techniques based on previous studies.

Encapsulation Techniques		Rate of Sub-Stitution (%)	Material of Encapsulated	Major Findings: (√) Improved Property, and (-) Unknown								
				Fracture Energy	Strengths and Elastic Modulus	Stiffness and Chloride Resistance	Condenses Index of Damage	Capillary Absorption and Permeability	Sorptivity Coefficient	Crack Width	Porosity and Surface Resistivity	Refs.
Poly styrene-divinylbenzene		0–2	Epoxy	√	-	√	-	√	-	√	-	[181]
Poly ureaf-ormaldehyde		1–4	Epoxy	-	√	-	√	-	-	√	-	[182]
Urea formaldehyde		0.25–2	Ca(NO_3_)_2_	√	√	-	-	-	-	√	√	[183,184,185]
		0–9	Epoxy	-			√	√	-	√	√	
Urea formaldehyde		0.5–5	Sodium silicate	√		√				√	-	[186]
Alginate		10	Ag^+^	-		√		√	√	√	-	
Poly-urea		0.8	Sodium silicate	-		-	-	-		√	-	[41]
Polyurethane		2.5–5	Sodium silicate	√	-	-	-	√		√	-	[141]
Melamine urea–formaldehyde		1–4	Epoxy	-	√	√	√	-	-	√	-	[187]
Polyvinyl alcohol		10	Calcium aluminate	-	-	√	√	-	-	√	-	[35]
Silica		5–10	Epoxy	√	-	-	-	√	√	√	√	[188]
Microcapsules		1–5	Bacterial spores	-	√	-	√	-		√	-	[156]
Poly-urea		0.25	Dicyclopenta-diene	-		√	-	-	-	√	-	[189]
		Content	Type of material	Ø_o_ (µm)		Length (mm)	Ø_i_ (µm)	Mixed		Thickness (µm)		
Capsule-based approach	Spherical	Cac_6_h_10_o_6_	Expanded clay	1000–4000		–	–	√		–		[119]
		Bacteria		1000–4000		–	–	√		–		[119]
		Na_2_FPO_3_		4000		–	–	√		–		[112]
		Tung oil	Gelatin	50		–	–	√		–		[190]
		Epoxy		50		–	–	√		–		[190]
		Acrylic resin		125–297		–	–	–		–		[13]
		Ca(OH)_2_		50		–	–	√		–		[190]
		Water	Paraffin	900		–	–	–		–		[179]
		Retarder agent	Wax	120		–	–	√		–		[48]
		Epoxy	UF	120		–	–	√		4		[191]
		Na_2_SiO_3_	PU	40–800		–	–	√		–		[124]
		Epoxy	UF	20–70		–	–	–		–		[13]
		Na_2_SiO_3_	Silica	5000		–	–	√		–		[115]
		Teb	Silica gel	4.15		–	–	√		–		[34]
	Cylindrical	Ca	Glass	1000		100	800	√		100		[31]
		Epoxy		7000		–	4000	–		–		[175]
		Pu	Ceramics	3000–4000		15–50	2500–3500	√		250		[115]
		Epoxy	Glass	5000		250	3000	√		–		[175]
		Epoxy		6000		250	4000	√		–		[175]
		Ca		–		75	800	√		–		[37]
		Bacteria		2200–3350		20–80	2000–3000	√		100		[122]
		Ca		2200–3350		20–80	2000–3000	√		100		[192]
		Ca		4000		200	3200	√		400		[193]
		Poly-acrylate		2200–3350		20–80	2000–3000	√		100		[192]
		Ca		–		100	3000	√		–		[37]
		Ca	Glass	4000		200	3200	√		400		[193]
		Epoxy		2200–3350		20–80	2000–3000	√		100		[192]
Vascular-based approach	Tubular and cementitious capsules	Epoxy	Glass	–		–	1500	√		–		[194]
		Foam		–		–	1500	√		–		[194]
		Ca		4000		–	3000	√		500		[37]
		Epoxy	Porous	25,000–35,000		–	–	√		–		[195]
		Ca	Glass	4000		–	3200	√		400		[193]
		Epoxy		2000		–	800	√		600		[13]
		Alkali silica		2000		–	800	√		600		[13]
		Epoxy		6000		–	4800	√		600		[196]
		Ca		–		–	1500	√		–		[194]
		Silicon		–		–	1500	√		–		[194]

## 6. Performance of SHC

Compressive strength [197,198,199] and durability [200,201,202] are the two most important properties of concrete. To assess the effectiveness of the self-healing process on hardened concrete properties, the effect of biomineralization on these properties must be determined. Crack, pore size, and distribution have a detrimental effect on the characteristics of concrete and, consequently, on the service life of concrete structures [44,203]. By taking advantage of the self-healing features, the durability of concrete may be increased by minimizing absorption, permeability, and diffusion, which are the primary modes of fluid and gas penetration into concrete [204]. Numerous investigations have been conducted to determine the effect of bio-based healing agents on the permeability and concrete absorption of water. As shown in Table 6, the inclusion of bio-based compounds reduces the permeability and water absorption of concrete structures. The effect of calcium carbonate precipitation on permeability was investigated with immobilized *Bacillus sphaericus* cells [122,205]. It was shown that specimens with bacteria immobilized in polyurethane had a sixfold decrease in permeability when compared to specimens without effective microorganisms. Additionally, the efficacy of immobilized *Bacillus sphaericus* in diatomaceous earth was revealed in terms of water absorption. The finding indicated that the absorbed water rate of specimens with immobilized bacteria was around 50% with respect to specimens without bacteria [67]. It has been observed that the addition of *Bacillus sphaericus* made the concrete more waterproof [206]. Over a 168 h period, a permeability test revealed that the coefficient of water absorption in treated specimens was six-times lower than in control specimens. The observed improvement in permeability may be attributed to the existence of bio-generated calcium carbonate as a result of bacterial metabolism. According to several previous studies, the biological method can significantly enhance the durability of concrete structures by sealing cracks and voids in a long-term manner. In contrast to the literature on durability, there is conflicting evidence about the effect of bio-based healing agents on concrete strength. It has been observed that using encapsulated *Bacillus sphaericus* in mortar reduces the compressive strength by 15% to 34% [69]; however, using *Bacillus sphaericus* in cube mortar increases the compressive strength at 7 and 28 days [207].

It was reported that a bio-based agent had a positive influence on compressive strength for the cell concentration of 5 × 10^6^ cells/mm^3^ [44]; however, the mortar experienced a reduction in compressive strength when the cell concentration increased to 5 × 10^8^ cells/mm^3^. The effect of *Sporosarcina pasteurii* on the compressive strength of mortar specimens was investigated after 7 and 28 days [208]. On the other hand, the highest concentration of immobilized *Sporosarcina pasteurii* on porous glass beads was shown to significantly boost the compressive strength of the mortar specimen by 24%. Furthermore, compressive strength increased as the cell concentration increased from 6.1 × 10^7^ to 3.1 × 10^9^ cells/cm^3^. In addition, the influence of immobilized ureolytic and denitrifying bacteria in protective materials on compressive strength has been documented [209].

**Table 6:** Influence of microbial agents on permeability, compressive strength, and water absorption.

Type of Microbial Agent	Influence on				
	Compressive Strength		Durability		Refs.
	Time (Day)	Influence	Water Absorption	Permeability	
*Bacillus sphaericus*	28	√	–	√	[207]
	7	√			
	7	√	√	–	[206]
	3	√			
	21	√			
	–	–	–	√	[69]
	90	x	–	√	[210]
	28	x			
	–	–	–	√	[122]
	–	–	√	–	[67]
*Bacillus sphaericus*	3	√	–	–	[211]
	7	√			
	28	√	√		[212]
*B. Pseudomycoides*	7	√	–	–	
	14	√	–	–	[64]
	28	√	–	–	
*S. pasteurii*	7	√	–	–	[208]
	28	√			
	28	√	√	–	
*Bacillus cohnii*	7	√	–	–	[213]
	56	√			
	28	√			
*Bacillus* *licheniformis*	3	√	–	–	[214]
	7	√	-		
	28	√	√		
*B. subtilis*	3	√	–	–	[70]
	7	√	–	–	
	28	√	√	√	
*Pasteurii bacteria*	28	√	√	√	[215]
	90	√	√	√	
*Diaphorobacter nitroreducens*	28	√	–	–	[209]
	7	√			
*B. megaterium*	–	√	–	–	[68]
	28	√	–	–	
*Bacillus pseudofirmus*	28	√	–	–	[119]
	7	√			
	3	√			
*B. sphaericus*	1	√	√	–	[73]
	7	√	√	–	
	28	√	√	–	

The findings indicated that the application of *Bacillus sphaericus* in concrete decreased the compressive strength at 7 and 28 days by 63% and 60%, respectively. It was also noted that although the utilization of denitrifying bacteria caused a reduction in compressive strength at both 7 and 28 days, immobilization of *Diaphorobacter nitroreducens* in expanded clay and granular activated carbon marginally enhanced the compressive strength. *Bacillus sphaericus* immobilization in metakaolin and zeolite, on the other hand, had a negative effect on compressive strength [216]. These inconsistent results may be explained by the brittleness of the calcium carbonate generated. Additionally, these discrepancies may have been caused by the use of various culture media and nutrients, as well as ambient circumstances. Apart from surface fractures, biomineralization can also be used to fill porosities and voids inside the concrete matrix [217,218]. Therefore, the application of microorganisms that are able to produce smaller bio-minerals may address the contradictory results for compressive strength.

## 7. Evaluation of Self-Healing Efficiency

**Figure 10:**
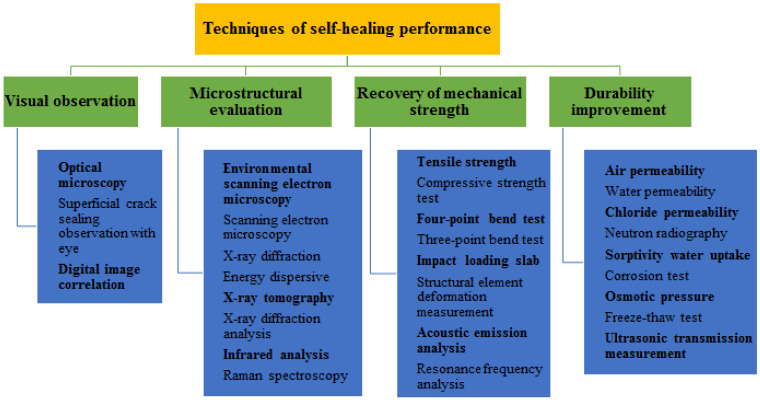
Techniques for measuring self-healing performance in concrete.

The review by Tittelboom and De Belie [219] provides an excellent summary of numerous strategies for evaluating self-healing performance. Image analysis and optical microscopy, scanning electron microscopy, ultrasonic transmission measurements, thin section analysis, X-ray radiography, digital image correlation, X-ray tomography, Raman spectroscopy, X-ray diffraction spectroscopy, and infrared analysis are some of the methods used to visualize crack healing, determine crack filling, and characterize the healing material [220]. Figure 10 depicts the techniques for assessing self-healing performance in concrete.

**Figure 11:**
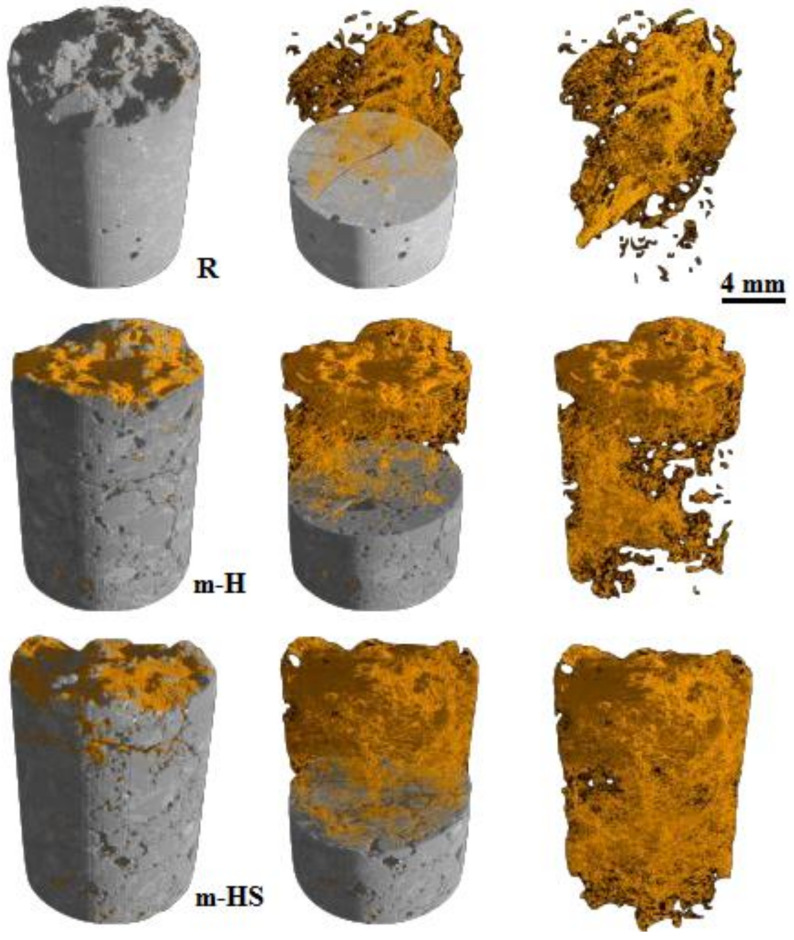
A 3D depiction of the healing products’ distribution in the control specimen (R) (brown color), the specimen with hydrogel bacterial spores (m-HS), and the specimen with pure hydrogel (m-H), after the process of self-healing. Left: surface of specimens in addition to the deposition; middle: dispersal of the deposits inside (Adapted with improvement from Wang [221]).

Figure 11 shows an example of employing 3D X-ray tomography to measure and visualize the distribution of healing products in a full sample [221]. The specimens containing hydrogel-encapsulated bacteria exhibited significantly higher precipitation (2.21%) than the reference (0.21%) and the pure hydrogel specimen. However, the majority of biogenic precipitation was seen to be dispersed on/in the surface layer. Water permeability tests at low or high pressure or by capillary water uptake can be used to check for losses in liquid tightness and water penetration can be visualized using X-ray or neutron radiography; in addition, air permeability can also be assessed. Moreover, corrosion testing, frost salt scaling, and chloride diffusion tests can all be used to determine liquid tightness. Furthermore, a compression test, tensile test, three- or four-point bending test, impact loading, acoustic emission study, or resonance frequency analysis can all be used to measure mechanical property recovery [222,223,224,225,226,227,228].

Another study reported the development of two test procedures for determining the sealing effectiveness [229]. The first test method determines the cement matrix’s ability to absorb water in the presence of a healed crack and compares it to the sorptivity of a sound specimen (best-case scenario) and to the sorptivity of a cracked and unhealed specimen (worst-case scenario) in order to determine the sealing efficiency. The test approach is identical to that described in EN 13057 [230], except that only a 40-mm-wide zone surrounding the fracture is exposed to water. The second approach determines whether the sealing products closing the crack can withstand a water flow under pressure (0.05–2 bar), which was provided via an interior hole in the specimen. If the specimen is not completely sealed, water will flow out and the amount will be monitored as a function of time. By comparing the water flow through the unhealed and healed cracks, the sealing efficiency can be defined.

### 7.1. Rupture Behavior

**Figure 12:**
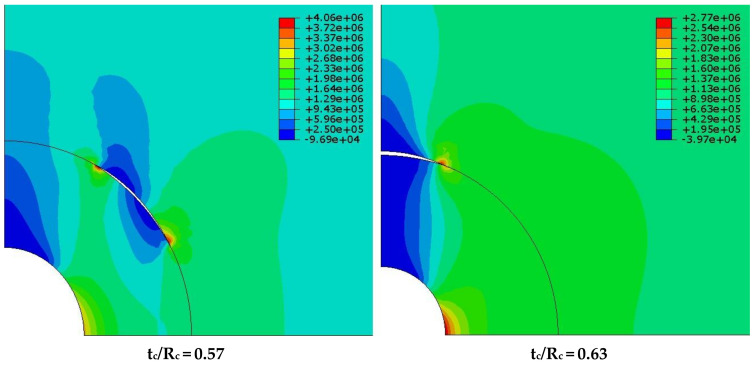
Onset of debonding: transition in the mode of initiation of the interfacial crack (color scale: maximum principal stress, in Pa) (Adapted with permission from MDPI [235]). Annotations: R_c_ = radius of half-length of the plate, t_c_ = the capsule wall thickness, and t_c_/R_c_ = geometrical ratio.

It is reported by Zemskov et al [231] that mechanical rupture is dependent on two factors: (1) the likelihood of a crack passing through a microcapsule, and (2) the microcapsule’s sensitivity to crack stresses [97,220]. Based on capsule morphology and amount in the bulk medium, numerical models show that the probability may be predicted [231]. Morphologies with a high aspect ratio, such as tubular geometries, have a higher probability of intersecting the crack plane. Additionally, elongated capsules can transport more healing agent to the crack surface for a given capsule dosage and volume [232]. Unfortunately, the cylindrical shape severely limits the applicability of cylindrical capsules, as high release efficiency requires capsules to be positioned perpendicular to the fracture plane. The mechanical parameters of the microcapsule, such as its strength and stiffness, also influence fracture propagation behavior in the capsule’s proximity [233]. When an inclusion has a higher elastic modulus than the surrounding matrix, it generates a stress field that deflects cracks away from the inclusion, whereas inclusions with a low elastic modulus attract cracks [234]. Once a crack successfully contacts a microcapsule, tensile forces concentrated at the crack tip must rupture the capsule. Strong interfacial bonding between the capsule shell and the cementitious matrix is required to prevent the crack from propagating along the interface, debonding the capsule, and preventing rupture [220]. According to numerical models, debonding is controlled by the bond strength, strength, and stiffness of capsules relative to the matrix, as well as the thickness-to-diameter ratio of microcapsules [235]. It is worth noting that these characteristics vary widely for very small elastic ratios (see Figure 12) [235]. This illustration may help to demonstrate the minimal interfacial strength necessary for self-healing applications when glass or ceramic capsules are used. While typical bonding between the capsule shell and cementitious matrix is actually unachievable, a rough microcapsule surface can aid bonding by increasing the mechanical interlocking and contact area for bonding to occur. This can be enhanced in particular polymeric shells by increasing the amount of surface deposition [187] or by utilizing shell prepolymers with a high molecular weight [97,220,233]. Alternatively, strong bonding may be generated by the use of a pozzolanic shell material; however, this possibility has not been fully researched. Additionally, silica-containing shells may react with the calcium hydroxide in concrete to generate a tight shell–cement contact [51].

### 7.2. Microcapsule and Macrocapsule Shell Robustness and Survivability

**Figure 13:**
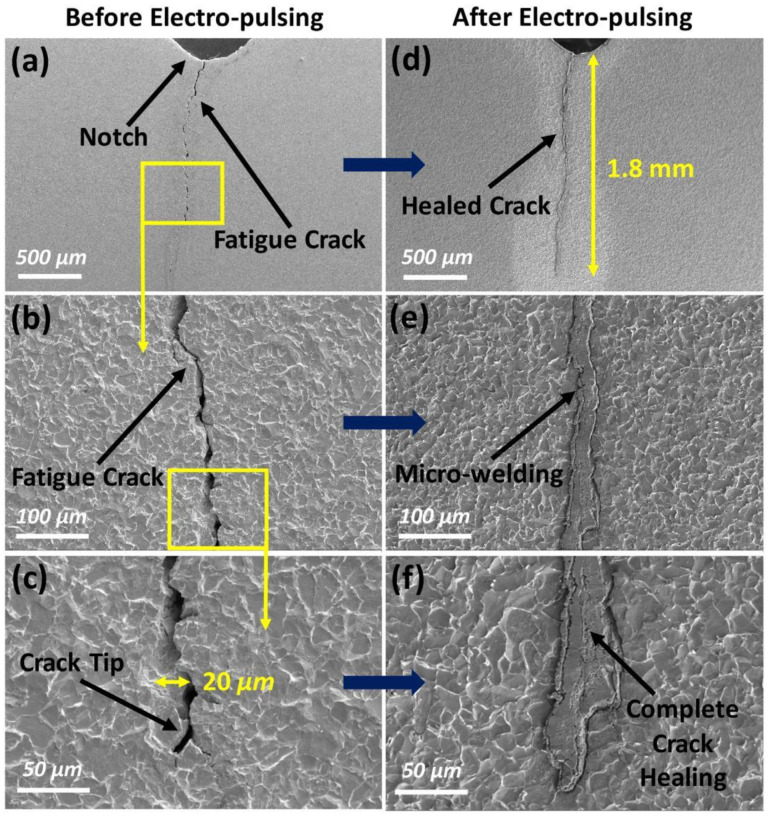
SEM micrograph of crack before and after the application of pulsed electric current (Adapted with permission from Elsevier [236]).

Microcapsule resilience is defined as the capacity of a microcapsule to tolerate high pressures and external stresses without losing its desired performance [141,220]. Microcapsules must be physically, thermally, and chemically resistant to guarantee that the healing agent is rapidly and effectively released into the damage zone when a crack occurs [41]. Most notably, mechanical robustness is required to prevent the early release of healing agents. This is because self-healing microcapsules are normally disseminated in the concrete preparation water during the mixing process [220]. Due to the brittle nature of the capsule shells, microcapsules are prone to damage or rupture as a result of collisions and the high shear forces imposed by mechanical mixing. Mechanical robustness is influenced by the microcapsule shell’s physical properties [187]. The maximal burst load of a microcapsule, in particular, is dependent on both the shell thickness and capsule diameter. Microcapsules with a low thickness-to-diameter ratio have a higher storage capacity per volume, but run the risk of early rupture and material diffusion through the shell before a crack occurs [159]. Increased shell material size improves the mechanical properties of microcapsules for survivability during the mixing process, but may obstruct mechanical release triggers [187]. Additionally, the shell morphology has a significant influence on capsule toughness, especially when a brittle material is used as a capsule shell [233]. In the case of thin tubes and microvascular systems, and to minimize their damage during mixing process, long, thin tubes and microvascular systems require meticulous preparation and protection during setup. Tubular capsules are frequently protected by fixing them to bars, adding a layer of mesh reinforcement above the capsules, and encasing them in cement mortar or wire wrappings [236]. The typical load–displacement behavior observed for both the self-healing and control beams is depicted in Figure 13. Before and after the application of a pulsed electric current (Figure 13a,b), the length and width of the crack are measured from these photos. The average pre-crack width is around 20 m, and the overall pre-crack length is approximately 1.8 mm (Figure 13c,d). The gap closes until the notch is reached after the application of the pulsed electric current, as seen in the SEM photos. The fracture, including the tip of the crack, was completely healed (Figure 13f). The crack opening at the notch shrank from 20 m to almost nothing, almost completely collapsing the crack. Microwelding could also be detected between the broken surfaces (Figure 13e). The pulsed electric current also altered the microstructure of the sample near the crack but had no effect on the microstructure of the sample far away from the break.

This is supported by the experimental results, which demonstrate that the specimen’s crack strength was 32% greater than that of the control specimen [220]. In summary, discrete spherical microcapsules are easier to integrate into concrete but supply only a limited amount of repairing material [179,220].

Furthermore, it is reported by Mullem et al. [237] that the use of macrocapsules in cement composites dates back to 1990s [2,31]. Since then, macrocapsules have been employed in cementitious materials to integrate a wide range of therapeutic medicines [40,193]. Glass tubular capsules filled with polyurethane have been used as macrocapsules [238]. Prior to casting, the glass capsules were inserted into the molds so that their location could be accurately controlled [239]. This had the advantage of requiring the healing agent to be applied only to the areas of the specimen where cracks were expected, avoiding the use of a healing agent with no healing potential [240]. The glass shell breaks when the cementitious matrix cracks at the position of a capsule, allowing the polymeric healing agent to flow out and patch the crack [237,241]. Several studies on the same sort of polyurethane enclosed in glass capsules have had positive results [2,31,40,193,237,241,242,243,244,245,246,247,248]. The bending strength of the polyurethane may be partially restored (up to 35%), and once hardened, it is capable of bridging moving cracks (i.e., fissures that vary in width due to changes in the load in the cross-section) with an extra crack opening of 50% to 100% [244]. In terms of capillary water absorption [244,245] and water permeability [242,243], the recovery in liquid tightness, which is commonly referred to as sealing efficiency, is very good—even flawless. In terms of resistance to chloride intrusion, an accelerated chloride diffusion test revealed that repaired cracks at a depth of 6 mm away from the exposed surface had a healing efficiency of 75% or higher [237]. Furthermore, a probabilistic service life forecast performed in the same study revealed that the first repair for an RC slab with this encapsulated healing agent in exposure class XS2 would be required after 60–94 years, rather than 7 years [247]. Non-steady-state chloride migration testing revealed that all tested materials had full durability recovery [248]. In a separate investigation, favorable performance in chloride conditions was also reported, with enhanced resistance to chloride-induced reinforcing corrosion [246]. In brief, the growing concern for structure safety and sustainability necessitates the application of innovative self-healing materials and preventive repair procedures. In Table 7, an overview is given of healing agents used for application inside SHC. For each agent, the most relevant properties are mentioned.

### 7.3. Recovery of Durability

**Figure 14:**
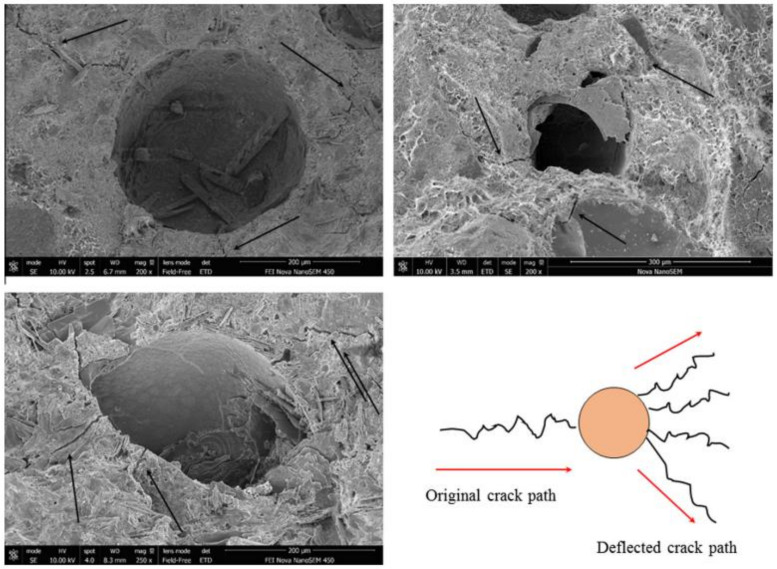
SEM images showing the crack propagation and deflection pattern around the microcapsules (Adapted with permission from Elsevier [256]).

It is well established that increasing the durability of cracked concrete by lowering the permeability of the damage zone by crack sealing or densification of the damaged cementitious matrix is possible [191,220]. In repaired mortar specimens containing epoxy capsules, a decrease in capillary porosity, continuous pore diameter, pore connectivity, and chloride penetration is found [255]. Moreover, larger microcapsules are more effective than small microcapsules when the same amount of healing substance is used [234]. For instance, using 230 µm capsules increased permeability resistance by around 22%, but using the same weight fraction of 132 µm capsules reduced impermeability by just 14% [256]. Elemental analysis revealed that the microcapsules and host matrix were mostly connected by ettringite and calcium silicate hydrates (C–S–H). This indicates that hydration around the polymeric shell remained unaffected, implying that no unfavorable interactions occurred. The proclivity of microcapsules to attract micro-cracks led to a similar pattern found in all samples containing microcapsules (see Figure 14). 

According to reports, closing toward the crack’s tip, the distance between the crack planes steadily decreases and the healing processes becomes more efficient. In addition, sodium silicate was shown to be more effective at sealing than some polymers. Complete sealing is seen in 110–170-µm-wide cracks and 80% sealing in 180–250-µm-wide cracks in specimens containing 6% of 290 µm sodium silicate microcapsules [256]. When 6% of 230 µm epoxy microcapsules are utilized, however, only 33% and 26% of cracks with a width of 110–170 µm and 180–250 µm are closed, respectively [234]. Because healing agents such as sodium silicate have limited interaction with non-cementitious aggregates, the impact of healing may be diminished if aggregates surround the microcapsules [41]. The application of viscosity-modifying chemicals, on the other hand, may give a solution for dispersing higher volume fractions of microcapsules, resulting in improved performance. Furthermore, higher sodium silicate content in the microcapsules may result in higher self-healing levels at lower microencapsulate concentrations.

## 8. Applications of SHC

The development of novel self-healing cementitious materials that mimic the behavior of natural living systems has attracted the global research community across a broad range of engineering and scientific disciplines and has the potential to revolutionize the way in which concrete structures are designed and constructed [7,37,257]. There are two types of self-healing in cementitious materials, either engineered (autonomous) or natural [15]. Calcite precipitating bacteria, microcapsules, and vascular networks holding healing agents and shape memory materials are some of the novel self-healing technologies that constitute the basis of the work researched.

Table 8 provides an overview of various technologies [257]. According to published research, there has been a lack of data on how self-healing cementitious materials may be used in the construction sector and which applications are most suited to them in terms of added value [257].

**Figure 15:**
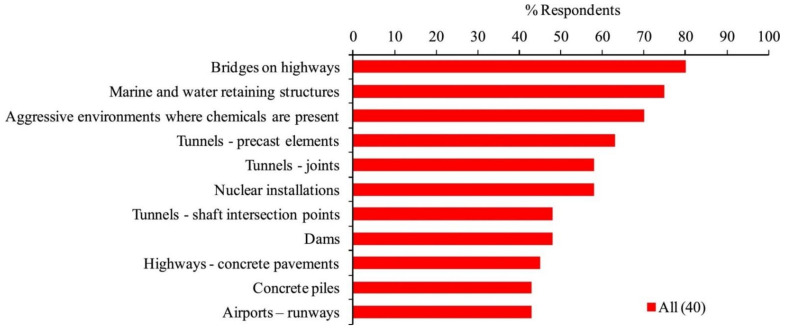
Applications for which SHC could have particular appeal (Adapted with permission from Elsevier [257]).

Figure 15 depicts a number of applications in which SHC may be particularly useful. The primary objective for these applications was to reduce maintenance costs and avoid water infiltration [97,165,257]. Bridges, marine/water-retaining structures in harsh environments, tunnels, and nuclear sites were the most perceived applications when presented with a list prepared during the initial part of the market study [257]. Figure 15 shows the replies, which were mentioned by more than 40% in each case. When respondents were also asked to freely remark on any specific application or aspect of the structure that might fit self-healing cementitious materials, it was found that the responders offered a wide range of options, including several that were repeated from the suggested list [257]. Tunnel joints and linings were the most often indicated applications, cited by one-third of responders and one-quarter of design team members. In addition, difficult-to-reach places, the nuclear industry, water-retaining buildings, and airports were among the numerous suggested applications [7,37,257].

## 9. A Preliminary View about the Costs of the SHC Technology

The technology will be available for the first time this year. It will be available in three different forms: self-healing concrete, repair mortar, and liquid repair medium. Unfortunately, the technology is currently extremely expensive, costing approximately USD 33–44/m^2^ [262]. As a result, it will initially be viable only for projects where leakage and corrosion are very severe, such as underground and undersea constructions. The cost of the calcium lactate required for the bacteria to manufacture calcite is a factor, but researchers are striving to develop a less expensive, sugar-based alternative worldwide, and as demand for concrete grows, the price should fall. However, SHC to regain mechanical properties is a realistic approach for increasing concrete durability by cutting maintenance and repair costs for concrete buildings. Bridge maintenance costs an estimated USD 5.2 billion each year, according to some estimates [128]. Furthermore, structural maintenance increases capital loss (due to traffic bottlenecks) and productivity, which is projected to be 10 times the cost of erecting a structure in the United States [263]. Owners of concrete structures spend an estimated USD 18–21 billion per year on upkeep, protection, and repair [264]. Since 1998, the American Society of Civil Engineers (ASCE) has been reported to have invested USD 3.6 trillion in the repair and maintenance of concrete structures in the United States [265]. In the United States, around 45% of construction investment is spent on inspection, maintenance, and repair, but in Europe, this expenditure is 50%; moreover, China invests USD 39.34 billion per year in corrosion protection for reinforced concrete structures [128]. As a result, integrating healing agents into concrete can minimize structural repair and maintenance costs and produce more durable and cost-effective structures.

## 10. Limitations and Hotspot Research Topics for Future Investigations

Currently, the application of self-healing technology is still inconsistent, in part owing to a lack of systematic data reporting and defined testing and characterization criteria [7,231]. This entails identifying (1) the desired concrete property to be regained during healing, (2) the desired crack type to be healed, (3) test procedures that accurately imitate crack initiation and propagation [235], and (4) suitable test methods for estimating the required concrete property recovery. The ‘self-healing’ ability of concrete is divided into two categories: self-sealing and self-healing of cracks [115,117]. Sealing refers to the obliteration of openings, whereas healing means the restoration of the essential mechanical properties of concrete. It is critical to distinguish between these two concepts because the requirements for durability-based and strength-based design are not the same [158]. The function of a concrete construction or exposure circumstances might determine which properties are required for recovery. For example, while crack sealing is more beneficial for concrete buildings exposed to chlorides than for crack healing, such as concrete facilities near coastlines and factories, interior structural elements are less likely to benefit from crack sealing compared to the healing process [60]. Accordingly, the selection and optimization of microcapsules depend on the prescribed healing [41,141]. For this reason, microcapsule selection and optimization depend on the recommended healing. Crack widths range from a few microns to several centimeters. Since a single microcapsule cannot be designed that can be utilized to heal cracks of different sizes and levels, it must be designed to target cracks within a specific range of crack widths [185,256]. For example, due to autogenous, drying, and thermal shrinkage, early-age concrete is vulnerable to cracking. Deformations caused by autogenous shrinkage are substantially lower than those caused by drying shrinkage and thermal volume changes, generating different levels of cracks widths [266]. In most cases, the micro-cracks caused by drying are less than 0.1-mm-wide and penetrate no deeper than 18 mm [267]. Poor heat dissipation can lead to micro-cracks that are 0.01–0.1mm in width and with less than 50 mm penetration depth [268]. Throughout its service life, concrete is vulnerable to structural fissures as a result of loading. The size of these fissures varies greatly depending on the amount of stress they are subjected to. The Canadian Standards Association specifies that the maximum fracture width for reinforced flexural elements is 0.33–0.4 mm, based on its being an exterior or interior crack [269]. When it comes to repairing micro-cracks (no wider than 0.2 mm), microcapsules appear to be the best option, and the self-healing mechanism should focus on the most critical crack in order to acquire the best results [1]. Simulating genuine crack processes is important to appropriately estimate the healing capacity [46]. However, most research on self-healing does not take into account the method used to cause cracking in order to determine the crack’s features [220]. Compression tests, indirect tensile tests, and flexural bending [82] are commonly used to generate cracks [97]. Three- and four-point bending produce V-shaped cracks that are broad at the crack opening but taper to a point at the fracture’s termination [12,220]. Split tensile tests, on the other hand, yield cracks that are more consistent in breadth throughout their length. Three- or four-point bending fractures are indicative of flexural or shrinkage cracks in reality, whereas tensile testing can mimic interior cracks induced by constrained deformations [140]. Many studies use varied techniques and assessment criteria to examine self-healing due to a lack of standardization. Mechanical strength recovery is usually measured by comparing mechanical characteristics between pre- and post-healing specimens [117], or between healed and non-healed specimens under the same curing circumstances [35,69]. The recovery of durability is measured in terms of liquid permeability, sorptivity gas, permeability, and chloride diffusion [128].

The target of the current study is to show that bacteria-based self-healing (BSH) cementitious composites can ‘self-heal’ by successfully sealing cracks that occur within a particular width range [9,154,270]. Moreover, current research primarily focuses on a laboratory scale, with very few outdoor-scale trials [7,9,58,97,118,119,122,165,221,270]. This has resulted in most experimental work being conducted on mortars and not on concrete, where the behavior is likely to be different due to the influence of the aggregates and differing crack patterns. Based on this comprehensive review, several hotspot research topics for future research can be successfully recommended as follows:

− Strategies to enhance bio self-healing and lower costs will bring about a shift in contractors’ acceptance of bio-concrete as a material of choice in the near future [271].
− Future research should concentrate on the protection of bacteria in their natural habitats and the maintenance of a constant supply of nutrients.
− The area of study should be extended to include the impact of healing on the return of these BSH materials’ original mechanical characteristics.
− It is imperative that further study be done to see how these BSH systems perform in real environmental conditions, such as when the concrete is older, when it has many cracks, or when it is subjected to varied sustained loads.
− There is still a shortage of large-scale meaningful commercial experience, where BSH strategies have not been employed in practical engineering projects till now.
− Additional research is needed to determine how to reduce related expenses, specifically microorganisms, nutrients, and labor [44,272].
− A comprehensive evaluation of whether healed BSH concrete elements will achieve a similar or equivalent lifetime performance when compared to uncracked conventional concrete elements would require long-term durability tests to be conducted.
− Further research is needed to determine how to cheaply scale up the various processes involved in the manufacturing of BSH materials.
− Despite recent advances in the design of protocols for bio-based self-healing concrete, the available research is still hindered by a lack of numerical simulation, which would allow them to minimize experimental costs and time in the early stages of commercial application [273].
− In addition, it is required to establish methodologies, such as life-cycle assessments, to evaluate the cradle-to-gate sustainability of various BSH options.
− Further research is needed to expand the available bacterial isolates for case-specific bespoke solutions.
− In order to improve our understanding of the precise determinants underpinning an ideal bacterium, it could be better to use genetically modified microorganisms to aid in the focused selection of the most appropriate species.
− Despite this, SHC’s application to the concrete industry is still a while away.
− The feasibility of applying a healing agent during the mixing process and the persistence of bacteria in cured concrete require more investigation [44].
− It is critical that future research be directed toward the creation of capsules that are capable of surviving the concrete mixing and manufacturing processes without affecting the mechanical qualities of the resulting concrete.

## 11. Future Prospects

In the last two decades, there has been a surge in interest in self-healing materials, particularly self-healing characteristics in cementitious materials, with dozens of researchers proposing various methods [7,274]. It is also not yet feasible to forecast which approach will outperform the competition in detail. Recently, many studies have been devoted primarily to autogenous healing; as a result, there has been a dearth of studies utilizing autonomous healing (capsule and vascular-based self-healing approaches) [128]. This is due to several factors, which are as follows:

− Autogenous healing is still limited to small cracks, and its reliability still is lower because it is dependent on the matrix composition at the time of crack development, which determines the possible reaction mechanisms.
− It should also consider the chance of a fracture reaching a capsule, the release efficiency, and the healable crack volume.
− Because tubular capsules must be manually inserted, this method is only suitable to precast concrete elements.

A comprehensive assessment of SHC research, reported by Tittelboom and Belie [7] showed that it is a highly transdisciplinary field that includes microbiology, chemistry, material science, civil engineering, and other disciplines. For a self-healing strategy to be effective, researchers from different disciplines must collaborate and communicate with each other [7]. Although any self-healing approach is possible to be used in situ, the key factor influencing its adoption and popularity will be the associated costs. As a result, it is critical to keep the costs of the mechanism as low as is feasible; ideally, they should be lower than the sum of the direct and indirect repair costs that occur over the structure’s service life. This may assist in persuading both designers and manufacturers to incorporate SHC into the design and construction of environmentally friendly and sustainable building systems. Concrete buildings will no longer be subject to wear and tear thanks to SHC. Scientists are approaching SHC in a variety of ways, despite the fact that it is still under development. Sodium silicate, fungi, and bacteria have all been used to manufacture some of these healing substances [97,198,275].

As reported by García et al [276], it is found that the need to provide lasting concrete constructions under extreme environmental and/or operating circumstances has driven the development of self-healing high-performance concrete (HPC), or even UHPC [276]. In order to produce long-lasting concrete structures, the concept is built on the merging of two breakthrough technologies, HPC and autonomous self-healing concretes. Due to the extreme conditions under which concrete is expected to last in the future, such as high mechanical fatigue and extreme temperatures, concrete developed in the future will be expected to survive for longer. Indeed, several new infrastructure projects will require long service lifespans that frequently exceed those specified in standards, such as concretes used in offshore or underground structures (where large temperature gradients and high pressure are expected), or concrete structures installed along coastlines (high chloride content), in subarctic or arctic regions, such as at low temperatures and ice abrasion, or in desert regions, such as at high temperatures and during fire conditions. In the majority of these scenarios, service life greater than 100 years will be necessary, although these periods will be much longer than the existing standard service life design. Because the service life can be extended more cost-effectively with a durable original design than with future rehabilitation, self-healing HPC will significantly lower maintenance costs in these unique conditions, despite the fact that they are predicted to raise the infrastructure’s initial cost [97]. Based on this review, it was noted that, in order to better characterize self-healing cement-based materials in general, not just the high-performance ones, a unified set of applicable, trustworthy, and accurate evaluation criteria is required to better characterize the self-healing techniques created. Nonetheless, given the tremendous gains that have already been documented, the self-healing HPC will likely be used in many concrete structures in the near future. After a few years, it will be easy to see why SHC is such a game-changer. It will allow builders to construct structures without having to worry about damage or extensive maintenance. SHC will benefit not only structures, but also sidewalks, as it is a fantastic solution for both of these problems. In cities and suburbs, smooth pavements can be built without concern for wear and tear. SHC, on the other hand, is still in the process of being perfected. While encouraging a designer to use a certain SHC may be difficult at the moment, it is expected to dominate the market within the next several years. Meanwhile, there are recipes for making our own SHC. One of the most widely used building materials is altering how we build and design our infrastructures once again.

## 12. The Role and Potential of Nanotechnologies as Innovative Solutions for Future Building Applications

Nanotechnologies have the ability to open the door to a brand new universe of building construction. Although the reproduction of natural systems is one of the most promising areas of modern technology, scholars are still struggling to comprehend their incredible intricacies [277]. They are also considered as new technologies that can improve the strength, lifespan, durability and resistance of building materials, as well as to provide the required properties. Moreover, nanotechnologies can decrease manufacture, retrofitting, and maintenance costs in the construction industry [278]. The potential of nanotechnologies to boost the effectiveness of construction materials, particularly concrete, which has the largest consumption, is substantial, leading to the creation of concrete composites based on the improvement of concrete’s mechanical properties [279]. Nanotechnologies can highly contribute to the development of concrete self-healing technology through the design and synthesis of capsules, production nanomaterials having the ability to heal concrete [280]. The University of Missouri has created a meta-material cloak that can be used to transport energy waves caused by tsunamis or earthquakes over materials such as plastics and steel to reduce the impact of shockwaves on buildings [281]. Although the widespread application of these materials is several years away, nanomaterial-reinforced concrete is a technology on the horizon. Aside from concrete, nanoparticles can be mixed with other materials to boost the effectiveness of road structural layers [282], boosting the stability of road systems, limiting the number of potholes, and decreasing the amount of repairs needed. Other sophisticated applications of nanotechnologies in building construction include [283]: (1) the manufacturing of low-cost corrosion-free steel; (2) a tenfold rise in thermal insulation materials; and (3) the production of self-cleaning coatings and films. Aside from buildings and infrastructure, nanotechnologies have the potential to improve the resilience of vehicles, particularly aircraft, in addition to an improved strength-to-weight ratio, and lightning protection is also among the advancements. Natural phenomena-related crashes can be reduced by better-built airplanes. Moreover, nano-engineered fire-resistant coatings that reduce the flammability and combustibility of the current polymers used in automobiles and structures are being developed [284]. The RILEM-197 [285] has reported that nanotechnologies in building materials are the first to synthesize in a clear manner the promise of nanotechnologies in terms of building construction development, namely [277]:

- The manufacturing of low-cost corrosion-free steel.
- The use of nanoparticles, carbon nanotubes, and nanofibers to boost the strength and durability of cement materials on the SHC capability of high-performance concrete, as well as to reduce pollution [280].
- The creation of coatings and thin films with self-cleaning and self-coloring properties to reduce energy usage.
- The production of thermal insulating materials with ten times the performance of current market choices.
- The creation of nanosensors and nanomaterials with sensing and SHC capabilities.
- Further study in the realm of nanotoxicity is also required; nevertheless, significant caution should be utilized while employing nanoparticles [277].

## 13. Conclusions

Self-healing of concrete is a complicated process that involves a combination of physical, chemical, and mechanical forces. Due to the low tensile strength of concrete, cracks are a regular occurrence. These fissures reduce the durability of concrete by providing a convenient conduit for the passage of liquids and gases that may contain dangerous compounds. If micro-cracks become large enough to reach the reinforcement, not only will the concrete be harmed, but the reinforcement will also deteriorate. As a result, it is critical to keep the crack width under control and to cure the cracks as quickly as feasible. The creation of SHC is the subject of this research since the costs of maintaining and repairing concrete structures are often significant. Self-healing concrete cracks would extend the lifespan of concrete structures, making the material slightly more resilient but also more efficient. SHC is a technique that can be used to create smart materials and a high degree of flexibility. Depending on the application, multiple self-healing concrete technologies can be used. The most challenging issues for all self-healing technologies in the concrete industry are general adoption, increased expenses, and long-term durability effectiveness confirmation.

Restriction of crack initiation, wet–dry cycle, the use of SCMs such as GGBS, silica fume, and fly ash, and the use of expansive minerals such as bentonite clay, MgO, CSA, quicklime, and crystalizing mineral agents could all improve concrete’s autogenous self-healing potential. The success of autogenous self-healing, on the other hand, is heavily reliant on the amount of unhydrated cement or mineral left in the concrete. This has hitherto been limited to lower healable crack widths, longer healing times, and strength return. In contrast to autogenous healing, autonomic healing in concrete necessitates the discharge of the self-healing stimulating agent from restricted encapsulation or a continuous supply network. This is to increase the efficiency of concrete’s self-healing compared to the autogenous healing process. Microencapsulation, microvascular, and pellets containing various autonomic healing agents such as epoxies, methyl methacrylate, cyanoacrylates, microorganisms, minerals, and alkali–silica solutions are popular autonomic self-healing systems.

Encapsulation of biological healing agents in concrete is a superior alternative since the reaction between unhydrated cement particles and healing agents produces favorable effects. The effectiveness of a healing procedure, according to available healing techniques, is determined as to how much the matrix and healing agent interact with one another. The size and geometry of the crack also have a part in determining the efficient outcomes of concrete healing. For instance, mending uniform-width cracks differs from healing variable-width cracks. The ability of SHC to heal reduces the need for external intervention in locating and repairing internal damage (e.g., cracks). This results in concrete deterioration and reinforcing corrosion, as well as decreased costs and increased durability. The tactics, influential factors, mechanisms, and efficacy of self-healing were rigorously reviewed in this study. This research study also includes critical synopses of the characteristics, performance, and evaluation of SHC composites’ self-healing efficiency. Furthermore, development study is trending toward a broad grasp of SHC’s application potentiality as a superior concrete material and a turning point for fostering sustainable and serviceable concrete nanocomposites for modern buildings.

Based on this detailed literature review study, the development of self-healing high-performance concretes is a unique, cutting-edge, accessible, and environment-friendly concrete technology for proposing robust and sustainable homes for upcoming generations. It has been discovered that the initial strength attributes have yet to be recovered. Because only partial recovery of strength properties after fracture healing is possible in conventional concrete, this element will be more difficult to achieve in high-strength concrete due to the greater initial mechanical properties. Furthermore, the healing of larger cracks must be attempted, which will be critical in particularly aggressive settings or when concrete structures are subjected to very aggressive operating circumstances. Both milestones must be met, which necessitates the adoption of self-healing systems that are well-suited to the concrete matrix. It has become evident that nanotechnology will be one of the primary techniques used to indicate that self-healing systems based on nanoparticles are likely to have a lower negative impact on the resulting initial strength qualities than those based on microcapsules. Furthermore, it is possible that SHC will allow builders to create structures without concern of damage or costly maintenance. There is also evidence that SHC is a truly interdisciplinary hotspot research topic that integrates civil engineering, chemistry, material science, microbiology, and other disciplines. Furthermore, the SHC’s limitations and future opportunities, as well as hotspot research issues for future studies, have been successfully highlighted.

## Figures and Tables

**Table 2:** Methods used to evaluate the crack’s healing efficiency.

Method		Possibilities	Refs.
Visualization and determination	X-ray radiography	Imagining release encapsulated agent from embedded capsule	[13]
	Scanning electron microscopy	Imagining crystal deposition	[16,47,67,111]
	Environmental scanning electron microscopy	Imagining breakage of partially embedded capsule	[16]
	Thin section analysis	Imagining crystal deposition inside crack	[95,112]
	Optical microscopy and image analysis	Imagining crystal deposition and determination of healing rate	[111,113,114]
	X-ray tomography	Imagining release encapsulated agent from embedded capsule in 3D	[115]
		Release of encapsulated agent	[13,115]
	Environmental scanning electron microscopy	Imagining breakage of partially embedded capsule	[16]
	X-ray diffraction analysis	Finding of crystalline materials	[116]
		Determination of crystalline materials	[117]
	Infrared analysis	Finding of precipitated products	[118,119]
	Raman spectroscopy	Determination chemical composition	[111]
	Correlation of digital image	Crack tends to close after treatment	[72]
	Micromorphology	Crystals starts to deposit in crack	[95,112]
	Image analysis/optical microscopy	Determination of healing rate	[113,114]
Regain tightness	Air permeability	Air flow via crack	[34]
	Water permeability	Water flow via crack	[90,115,120,121,122]
	Capillary water uptake	Capillary water uptake by crack	[112,123]
	Corrosion test	Resistance against corrosion	[67,124]
		Corrosion resistance	[124]
	Neutron radiography	Visualize capillary water uptake	[53]
	Frost salt scaling	Resistance against frost salt scaling	[125]
	Ultrasonic transmission measurements	Continuity of material	[117]
	Osmotic pressure	Resistance against ion ingress	[117]
	Chloride diffusion	Resistance against chloride ingress	[117]
	Pressure water/air permeability	Water flows via cracks healed	[115,122,126,127]
	Salt scaling	Salt scaling resistance	[125]
	Ultrasonic transmission technique	Continuation of material	[117]
	Osmotic pressure	Ion ingression resistance	[128]
	Neutron radiography	Finding of water uptake	[53,129]
	Diffusion of chloride	Chloride ingression resistance	[130,131]
Regain mechanical properties	Water uptake through capillary action	Water uptake	[112,123]
	4-point bending test	Reopening of old cracks versus creation of new cracks	[115]
	3-point bending test		[2,37,95,132]
		Regain of stiffness and strength	[2,37,95,115]
	Resonance frequency analysis	Regain of stiffness	[20,51,105,120]
	Compression test and tensile test	Regain of strength, energy, and stiffness	[34,51,111,113]
		Regain of stiffness and strength	[34,51,133]
	Impact loading on slab		[128]
	Column/frame deformation		
	Frequency analysis	Capsule containing healing agent breaks	[20,105,116,120]

**Table 3:** Development in autogenous self-healing of concretes with the addition of mineral additives.

Mineral Additives	Ratio of Mineral Additives	Curing Condition	Type of Damage	Performance of Healing Crack Width in Time	Refs.
Crystalline additive (CA), alcium sulfoa- luminate (CSA), and FA	1.5% CA and OPC with 10% CSA	Water	Sp. Tensile test	-100–400 μm in 56 d Calcite	[25]
MgO	5% as cement replacement	Water	Applied load corresponding to 80% of the ultimate compressive strength for 7 days	-FTIR spectra analysis confirmed sharp bands around 1400 and 1500 cm^−1^ due to the stretching of C–O bonds, which corresponded to (CaCO_3_ and MgCO_3_) phases at 28 days - Cracks were sealed within 14 days	[148]
CSA	4.4%, 15.2% of cement	Flow water	Tension force	100 µm less flow	[149]
Superabsorbent polymers (SAPs)	0.5, 1%	90% RH and at a temperature of 20 ± 2 °C	Four-point bending test	-Cracks smaller than 150 µm almost completely healed -Cracks larger than 200 µm showed reduced visual closure	[150]
CSA, Mont.	Up to 10% (concrete)	Water	3PB, mechanical	-160–220 µm in 33 d Calcite, CASH	[24]
FA	15–20% with cement	Water	Shrinkage micro-cracks	-Meso-macro pores at 91, 182, and 364 d	[131]
FA, CA, SF	OPC + 10%SF, OPC + 1%CA OPC + 30%FA	Water	Splitting tensile test	-50 μm in 12 d, larger cracks heal proficiently with SF	[29]
Combination of SF and MgO	(5%SF + 5%MgO) As cement replacement	Water	Applied load corresponding to 80% of the ultimate compressive strength for 7 days	FTIR spectra analysis confirmed sharp bands around 980 cm^−1^ for Si–O bonds, which indicate C–S–H - Cracks were sealed within 14 days	[148]
Bentonite silica, Ca, CEA,	8% + up to 14%	Water, air, wet–dry, freeze–thaw	Compression, sp. Tensile	220 μm in 14 days Silica, bent, Ca	[26]
Slag, FA	30–40% of mortar	Water	Shrinkage	-Upgrade in strength	[151]
BFS	50% BFS + OPC	Water	Mechanical	It was 3 times quicker for cement	[29]
FA	5–155 of sand	Water	Freeze–thaw	-Increased damage by 90% in 1 day	[152]
Slag, FA, L	85% slag and 30, 50% FA; 50, 75	Water	3PB, mechanical	200 μm in 42 d	[27]
Bentonite, L, slag	2% PVA by vol. Dia = 40 μm Length = 8 mm	Water, wet–dry cycle, air	4 PB	-Naloclay advances the reloading bending capacity	[153]
Blast-furnace slag	66% as cement replacement	Saturated Ca(OH)_2_ solution	Micro-cracks	In 240 h of healing time, cracks of 10 and 30μm in width were healed by around 60% and 30%, respectively.	[142]
Bentonite	Nanoclay as internal water reservoir	Water	Mechanical	-Enhanced hydration for self-healing	[154]
MgO	4–12% of cement	Water	Drying shrinkage, 3PB	<500 μm in 28 d -Durability improved	[23,155]
CSA	PVA, up to 10% of mortar, 1:3	Water	3PB	100–200 μm-14 d,<100 μm-11 d, >200 μm-16 d	[156]
Clay lwas	Sodium mono fluorophosphate (Na-MFP) and PC-coated (Mortar)	Water	Mechanical	-Absorption decrease phosphorous, sodium, and fluoride, CH	[112]
CSA	Aggregates	-Water -Curing room (95–98% RH and 20 ± 2 °C)	Several CSA were broken into halves and pieced together	-The effective healing area reaches up to 1/3 of the fracture area after 3 months	[157]
Slag	66% of cement	Ca(OH)_2_ solution	Sliced, mechanical	60% of 10 μm in 10 days -Hydrogenate, C-S-H, ettringite	[142]
CA: microsilica + sand + cement	1–2% of cement	Water, open air	4PB	60% cracks sealed at ambient temperature	[158]
FA, quicklime	3% of cement	Water	Mechanical	Improved SiO_2_ solubility	[28]

Annotations: (H) Hauyne, (A) Anhydrite, (DME) Dynamic modulus of elasticity, (L) Lime/limestone powder, (CEA) Chemical expansive agent, (CSA) Carbonated steel slag, (FTIR) Fourier Transform Infrared Spectroscopy.

**Table 4:** Some of the bacteria encapsulated for SHC.

Type of Bacteria	Formation of Self-Healing	Main Findings	Refs.
*Bacillus cohnii*	C_6_H_10_CaO_6_	- Bacteria remain active for a half-year.	[121]
		- Seals larger cracks.	
*Bacillus subtilis*	C_6_H_10_CaO_6_	- Improves healing action.	[76]
*Bacillus*	Alteration of origin of Ca to carbonate	- Bacteria continue to be active for 120 days.	[76]
		- Origin of Ca affects healing percentage.	
*Bacillus sphaericus*	Ureolytic precipitation of Ca(NO_3_)_2_	- Increases strength.	[122]
		- Increases self-healing phenomenon.	
		- Decreases permeability.	
		- Improves healing ratio.	[67]
		- Condenses in permeability.	[35]
		- Diminishes water absorption.	
		- Lessens permeability.	[69]
*B. pseudomycoides*	Ureolytic activity	- Check deeper part of the crack.	[64]
		- Heals the crack width.	
		- Enhances the concrete matrices.	
*Bacillus subtilis*	Urea–2 CaCl_2_ curing	- Increases the compressive strength.	[65]
		- Increases the porosity.	
		- Enhances the permeability and water absorption.	
*Bacillus sphaericus*	Ureolytic precipitation of Ca(NO_3_)_2,_ 4H_2_O	- Reduces the water absorption.	[67]
		- Reduces the crack width.	
		- Increases strength.	
*Bacillus megaterium*	Urea yeast extract, CaCO_3_	- Heals the crack width.	[68]
		- Increases strength.	
		- Decreases permeability.	
*Bacillus sphaericus*	Ureolytic precipitation of Ca(NO_3_)_2,_ 4H_2_O	- Improves self-healing efficiency.	[69]
		- Fills larger cracks.	
		- Reduces permeability.	
*Bacillus subtilis*	Urea caco_3_ crystals, yeast extracts, NaCl	- Achieves higher strength recovery.	[70]
		- Increases strength.	
		- Heals the crack width.	
*Bacillus sphaericus*	Urea, calcium nitrate, urea (CO(NH_2_)_2_)	- Reduces permeability.	[35]
		- Optimizes the dosage of the microcapsules.	
		- Improves healing ratio.	
*Lysinibacillus sphaericus*	Urea Ca_2_+ ion, CaCl_2_ usage	- Increases the mortar properties.	[73]
		- Reduces porosity and permeability.	
		- Increases strength.	

**Table 7:** Summary of the healing agents based on previous studies.

Agent	Expansion		Components Number		Time of Curing	Curing Method	Strength (MPa)	Viscosity (mPas)	Refs.
	No	Yes	1	Less than 2					
Silicone	√	–	√	–	–	Air	–	–	[194]
Epoxy	√	–	√	–	–	Moisture, air	25	–	[249]
	√	–	√	–	60 °C, <100 min	Moisture, air, heat	–	–	[250]
Tung oil	√	–	√	–	–	Air	–	–	[159]
Epoxy	√	–	–	√	30 min	Contact component	5.1	150	[192]
PU and bacterial solution	–	√	–	√	–		–	600	[122]
Epoxy	√	–	–	√	±1 h		–	–	[159]
	√	–	–	√	40 min		45	360	[192]
	√	–	–	√	30 min		4.2	80	[192]
Na_2_SiO_3_ solution	√	–	√	–	–	Ca(OH)_2_ matrix	–	–	[251]
Epoxy	√	–	√	–	–	Moist, air	22	250–500	[175]
Alkali silica	√	–	√	–	–	Air	–	–	[13]
Methyl methacrylate (MMA)	√	–	–	√	–	Contact component	–	±1	[34]
	√	–	–	√	30 min	Contact component	50–75	±1	
	√	–	√	–	–	Heat	–	–	[252]
	√	–	–	√	1 h		50	34	[253]
PU	-	√	√	–	40–180 min	Moist	–	7200	[192]
		√	–	√	50–300 s	Contact component	–	600	[192]
	√	–	√	–	100 days	Water and O_2_	–	–	[16]
Polyacrylate	√	–	–	√	40 s	Contact component	–	7	[192]
Ca(OH)_2_ solution	√	–	√	–	–	CO_2_ in air	–	–	[159]
	√	–	√	–	–	Matrix	–	–	[254]
Bacterial solution	√	–	–	√	–	Water	–	–	[67]
Epoxy	√	–	–	√	–	Contact component	–	–	[13]
							
	√	–	–	√	–	Contact component	17.6	200	[191]
Foam	-	√	√	–	–	–	–	–	[194]
Na_2_FPO_3_ solution	√	–	√	–	28 days	Carbonation products	–	–	[112]

**Table 8:** Details of self-healing methods (Adapted with improvements from [257]).

Method	Scale	Type of Technology	Action of Healing	Requirements of Construction	Refs.
Bacteria	Micro or meso	Nutrient sources and bacterial spores are spread in a random pattern throughout the cementitious matrix.	In favorable conditions, spores are exposed to water and a nutrient source (i.e., on crack surface). Bacteria deposit CaCO_3_ on the surface of the crack.	As a normal component of the concrete mix, nutrients and bacterial spores are included.	[55,163]
Encapsulation	Micro	Direct mixing with water	Autogenous healing happens due the precipitation of calcium carbonate CaCO_3_ from decomposed urea into carbonate ions and calcium carbonate by bacteria in the presence of Ca^2+^present in atmosphere/hydrogel.	Melamine microcapsules/hydrogel containing cells of *Bacillus sphaericus* spores with yeast extract, urea, and Ca (NO_3_)_2_⋅4H_2_O.	[35,69]
Flow networks	All altogether	In a cementitious matrix, a small diameter hollow network is produced. Tubes containing a healing agent.	Concrete cracks will rupture the flow network, allowing the healing agent to enter the crack plane. The network supports recurrent damage/healing actions.	Prior to casting, the network is embedded in concrete, and the network forming tubes are removed 1 day later.	[9,154,257]
Nanomaterial additives	Nano	Direct mix	Refine micro-cracks by filling effect and pozzolanic reaction with Ca(OH)_2_.	Good distribution to prevent agglomeration.	[153,258]
Shape memory polymers	Meso or macro	PET strands in tendon form are anchored in the matrix. Post-tensioning strands are analogous in nature. An electric current is used to activate heat. PET shrinking results in a post-tensioning effect.	Cracks are repaired to the point where either natural autogenic healing or one of the numerous nano or micro scale healing techniques can take place.	Concrete placed into molds in a comparable way to a post-tensioning mechanism.	[72,116]
Coating method	Based on coating type	The coatings respond to changes in the pH of the solution or to the change in temperature and air, achieving an immediate response.	Chemical reaction/polymerization to form a tough, corrosion-resistant film	Based on coating type	[259,260,261]
Microcapsules	Nano or micro	Microcapsules are dispersed at random throughout the cementitious matrix.	A break propagates across the microcapsule, causing it to rupture. Healing agent is released into the crack plane.	Microcapsules will be included as a normal element of the mix of concrete.	[34,35,124,141,185]

## Data Availability

Data sharing not applicable.

## Abbreviations

SHC	Self-healing concrete
ASH	Autogenic self-healing
BSH	Bacteria-based self-healing
Ca(OH_)2_	Carbonation of calcium hydroxide
C-S-H	Calcium silicate hydrates
ECC	Engineered cementitious composite
HPC	High-performance concrete
PET	Polyethylene terephthalate
SAP	Superabsorbent polymer
SCM	Supplementary cementitious material
UHPC	Ultra-high-performance concrete
UHPFRC	Ultra-high-performance fiber-reinforced concrete
